# Researched Apps Used in Dementia Care for People Living With Dementia and Their Informal Caregivers: Systematic Review on App Features, Security, and Usability

**DOI:** 10.2196/46188

**Published:** 2023-10-12

**Authors:** Bing Ye, Charlene H Chu, Sayeh Bayat, Jessica Babineau, Tuck-Voon How, Alex Mihailidis

**Affiliations:** 1 Occupational Science and Occupational Therapy University of Toronto Toronto, ON Canada; 2 KITE Toronto Rehabilitation Institute Toronto, ON Canada; 3 Lawrence S Bloomberg Faculty of Nursing University of Toronto Toronto, ON Canada; 4 Department of Biomedical Engineering University of Calgary Calgary, AB Canada; 5 University Health Network Toronto, ON Canada

**Keywords:** mobile technologies, mobile apps, dementia, privacy, security, usability testing, informal caregivers, mobile phone

## Abstract

**Background:**

Studies have shown that mobile apps have the potential to serve as nonpharmacological interventions for dementia care, improving the quality of life of people living with dementia and their informal caregivers. However, little is known about the needs for and privacy aspects of these mobile apps in dementia care.

**Objective:**

This review seeks to understand the landscape of existing mobile apps in dementia care for people living with dementia and their caregivers with respect to app features, usability testing, privacy, and security.

**Methods:**

ACM Digital Library, Cochrane Central Register of Controlled Trials, Compendex, Embase, Inspec, Ovid MEDLINE, PsycINFO, and Scopus were searched. Studies were included if they included people with dementia living in the community, their informal caregivers, or both; focused on apps in dementia care using smartphones or tablet computers; and covered usability evaluation of the app. Records were independently screened, and 2 reviewers extracted the data. The Centre for Evidence-Based Medicine critical appraisal tool and Mixed Methods Appraisal Tool were used to assess the risk of bias in the included studies. Thematic synthesis was used, and the findings were summarized and tabulated based on each research aim.

**Results:**

Overall, 44 studies were included in this review, with 39 (89%) published after 2015. In total, 50 apps were included in the study, with more apps developed for people living with dementia as end users compared with caregivers. Most studies (27/44, 61%) used tablet computers. The most common app feature was cognitive stimulation. This review presented 9 app usability themes: user interface, physical considerations, screen size, interaction challenges, meeting user needs, lack of self-awareness of app needs, stigma, technological inexperience, and technical support. In total, 5 methods (questionnaires, interviews, observations, logging, and focus groups) were used to evaluate usability. There was little focus on the privacy and security aspects, including data transfer and protection, of mobile apps for people living with dementia.

**Conclusions:**

The limitations of this review include 1 reviewer conducting the full-text screening, its restriction to studies published in English, and the exclusion of apps that lacked empirical usability testing. As a result, there may be an incomplete representation of the available apps in the field of dementia care. However, this review highlights significant concerns related to the usability, privacy, and security of existing mobile apps for people living with dementia and their caregivers. The findings of this review provide a valuable framework to guide app developers and researchers in the areas of privacy policy development, app development strategies, and the importance of conducting thorough usability testing for their apps. By considering these factors, future work in this field can be advanced to enhance the quality and effectiveness of dementia care apps.

**Trial Registration:**

PROSPERO CRD42020216141; https://www.crd.york.ac.uk/prospero/display_record.php?RecordID=216141

**International Registered Report Identifier (IRRID):**

RR2-10.1159/000514838

## Introduction

### Background

Dementia is an irreversible condition that affects the human brain [1]. It often affects older adults [1] as the risk of developing dementia increases with age [2]. Dementia is ranked as the seventh leading cause of death worldwide [3] and the eighth leading cause of death in Canada [4]. People living with dementia often experience symptoms such as memory loss, disorientation, wandering, and behavioral and psychological symptoms of dementia (eg, agitation, aggression, and delusions) [1,5]. As such, people living with dementia gradually lose their independence, and many rely on the care provided by their family members or friends as dementia progresses [6-8]. These care providers are usually referred to as informal caregivers [9].

The literature indicates that people living with dementia would like to remain at home [10]. Staying at home allows people living with dementia to remain more independent with self-autonomy, thus improving their quality of life (QOL) [11,12] and reducing care costs [13]. However, research indicates that informal caregivers experience caregiver burden and require additional support for their daily caregiving duties [14,15]. Thus, it is urgent to find effective and accessible solutions to assist and support informal caregivers and people living with dementia in maintaining their QOL.

Mobile technology has become an essential part of our daily lives as it is widely used worldwide [16,17]. Studies indicate that mobile devices can support people living with dementia in their daily lives and improve the QOL of both people living with dementia and their informal caregivers [18-25]. For technology to be effective and acceptable, it needs to fulfill the users’ needs [25,26]. However, studies have demonstrated a gap in technology that meets users’ needs [27,28]. Studies have illustrated that privacy concerns could hinder technology adoption for people living with dementia and their informal caregivers. However, there is limited research on the ethical considerations for people living with dementia [29-32]. To address these concerns, it would be beneficial to understand the privacy and security issues that people living with dementia may face on mobile devices.

Mobile apps have been developed to assist with dementia care [18,19]. Previous literature reviews have examined apps for dementia medical screening [33,34] and the effects of the apps on informal caregivers [35]. However, little is known about the needs of people living with dementia and their informal caregivers regarding apps for dementia care [27,36]. This review advanced our knowledge of apps in dementia care with a focus on app features, usability, and acceptability of the apps for people living with dementia and their caregivers, as well as providing an analysis of safety and privacy agreements. To our knowledge, this is the first review to comprehensively examine the literature with a user-centered approach.

### Objectives

The major research aims were as follows:

1. To identify the types and features (ie, professional referral) of researched apps that are used to support dementia care (ie, QOL, dementia education, support and services, and performing activities of daily living [ADLs]) for people living with dementia and their informal caregivers
2. To identify privacy and safety features of researched apps from the perspective of end users (ie, concerns over being hacked) and developers and researchers (ie, apps with security prompts built in, privacy policies, or encrypted data)
3. To describe the methodologies that have been used for usability evaluation
4. To summarize the results obtained from the usability evaluation of the apps

## Methods

This systematic review was conducted according to the previously published review protocol in the *Gerontology* journal [37] and has been registered in PROSPERO (CRD42020216141). The review also follows PRISMA guidelines (Multimedia Appendix 1).

### Information Sources and Search Strategy

The search strategy was developed in consultation with and executed by an information specialist. It included using both text words and subject headings (eg, Medical Subject Headings and Emtree) in areas related to dementia and Alzheimer disease (AD) and mobile apps. The databases searched included ACM Digital Library, Cochrane Central Register of Controlled Trials (Ovid), Compendex (Engineering Village), Embase (Ovid), Inspec (Engineering Village), MEDLINE ALL (Ovid), the American Psychological Association PsycINFO (Ovid), and Scopus.

A manual search of the reference lists of the included studies and relevant systematic reviews was also conducted. All databases were searched from resource inception. Searches were limited to human participants and studies in the English language when possible. The full search strategies are presented in Multimedia Appendix 2.

### Study Selection

#### Overview

Duplicates from the initial search results were removed using the EndNote software (Clarivate Analytics) [38]. The remaining results were then imported to Covidence [39] for title, abstract, and full-text screening. In total, 2 researchers independently screened the titles and abstracts based on the inclusion and exclusion criteria. Disagreements were first discussed between the 2 researchers. A third researcher was involved if an agreement could not be reached. The remaining studies then underwent full-text screening by 1 researcher using the same inclusion and exclusion criteria. The decision to have a single reviewer conduct the full-text screening was due to time limitations and the limited availability of the reviewers, which made it challenging to conduct this dual-reviewer process. To avoid potential bias and ensure rigor in the screening process, several steps were taken. First, we used clear, predefined inclusion and exclusion criteria to guide the reviewer’s decision during full-text screening. Second, to minimize errors and ensure consistency, the reviewer sought a second opinion from the coauthors for any ambiguous cases encountered during full-text screening. In addition, the reviewer conducted a random sample check of the included studies to assess agreement. Although full-text screening only involved 1 reviewer, it is important to note that dual reviewers were involved during title and abstract screening to enhance accuracy and reduce bias.

#### Inclusion Criteria

Studies were included if all the inclusion criteria were met: (1) the studies included people living with dementia who lived in the community, their informal caregivers, or both; (2) the studies focused on apps used in dementia care; (3) the studies used smartphones, tablet computers, or handheld computers; and (4) the studies conducted a usability evaluation of the app with end users.

#### Exclusion Criteria

The exclusion criteria were as follows: (1) studies that involved nonhuman participants; (2) studies that included only healthy participants or people living with dementia who lived in a nursing home, a care facility, or long-term care; (3) studies that involved apps developed for health care professionals, such as nurses, physicians, therapists, clinicians, or professional caregivers; (4) studies that included apps focused on dementia cognition assessment or diagnoses; (5) studies focused only on a conceptual app, the description and design process, or app development without user evaluation; (6) review studies, such as literature reviews or systematic reviews; (7) studies that were not peer-reviewed, such as preprints; and (8) presentations, protocols, commentaries, abstracts, supplements, or posters.

### Summarized Aspects in Terms of App Evaluation

To ensure a comprehensive analysis related to app evaluation, specific aspects were investigated. Aspects included the screen size of mobile devices, app user interface, stigmatization while using the app, physical considerations of people living with dementia, technical support, and other relevant factors identified in the literature. These aspects were carefully selected based on their relevance to the research aim and their potential impact on the outcomes of interest. By considering these aspects, a broad range of factors could be identified that may influence the effectiveness and usability of mobile apps.

### Data Extraction

For each included study, information was extracted by 1 researcher and verified by a second researcher for accuracy and completeness. Discrepancies were discussed between the 2 researchers to reach a consensus. An Excel (Microsoft Corp) file was used for logging the extracted data, which included the following:

1. Publication details: authors, study title, publication year, publication type (ie, conference paper or journal paper), country of origin, and funding source
2. Study characteristics: study design (ie, qualitative or randomized controlled trial [RCT] study), data collection length, and study aim
3. Participant characteristics: participant type (ie, caregivers or people living with dementia), severity of dementia, sample size, participants’ age and gender, and participants’ previous experience with mobile technology or computer skills
4. Characteristics of mobile apps in dementia care: app name, language or languages offered, operating system (OS), app purpose (ie, reminders, reminiscence, and navigation), app features (such as professional referral), security and privacy features, device types used in the study (ie, smartphone and tablet), app support population (ie, people living with dementia or their informal caregivers), app commercialization (whether an app was commercialized), types of data entered by the users, and data collected by the app
5. Approach for usability evaluation (ie, survey or interview), measurement of usability evaluation (ie, System Usability Scale [SUS]), and relevant usability findings.

### Quality (Risk of Bias) Assessment

Study quality was assessed using two critical appraisal tools: (1) the Centre for Evidence-Based Medicine critical appraisal tool and (2) the Mixed Methods Appraisal Tool [40,41]. The case study research was assessed using the Centre for Evidence-Based Medicine critical appraisal tool. Mixed methods studies, qualitative and quantitative studies, RCTs, and non-RCTs were appraised using the Mixed Methods Appraisal Tool. The study quality assessment was completed by 1 researcher and checked by a second researcher. A major goal of this review was to summarize all available apps for dementia care. Therefore, all studies regardless of quality were included to add richness to our findings.

### Data Synthesis

A meta-analysis was not performed owing to the heterogeneity of study designs, mobile apps, participants, and outcome measures. For example, the participants involved in the studies were at varying stages of dementia. The mobile apps evaluated presented a wide range of features and functionalities. The data were tabulated, summarized, and grouped according to the 4 research aims. On the basis of the nature of the data and the research objectives, a thematic analysis was chosen [42]. Thematic analysis is a qualitative analysis method that allows for the identification of key themes. It allowed us to uncover the perspectives related to our research aims in a more meaningful manner. In addition, thematic analysis allowed us to analyze and interpret the qualitative data extracted from different sources, such as interviews and focus groups [42]. Therefore, we decided to use thematic analysis to generate themes through an iterative coding process. During open coding, the researcher identified initial patterns and concepts within the data. Clear criteria, including relevance, frequency, and consistency, were established to guide theme selection. Recurring patterns and concepts related to the research aims were identified and then grouped into themes and subthemes [42].

The coding process was completed by a single researcher and involved a structured sequence of steps. The researcher conducted the coding using NVivo, a qualitative data analysis software (released in March 2020; QSR International) [43]. The researcher first started a complete review of all the included papers, focusing particularly on the results sections, to establish familiarity with the data. The initial coding process included breaking down the textual content into meaningful nodes in NVivo. Each node was labeled with a description to capture the essence of the content. Throughout the process, the researcher constantly reviewed and compared the new nodes with the existing nodes to ensure the accuracy of each node. Similar nodes were grouped into broader categories to identify emerging patterns. To further ensure accuracy and consistency, the coauthors also checked the work to confirm that the derived themes were coherent and comprehensible. The entire coding process was completed manually within NVivo.

## Results

### Overview

**Figure 1:**
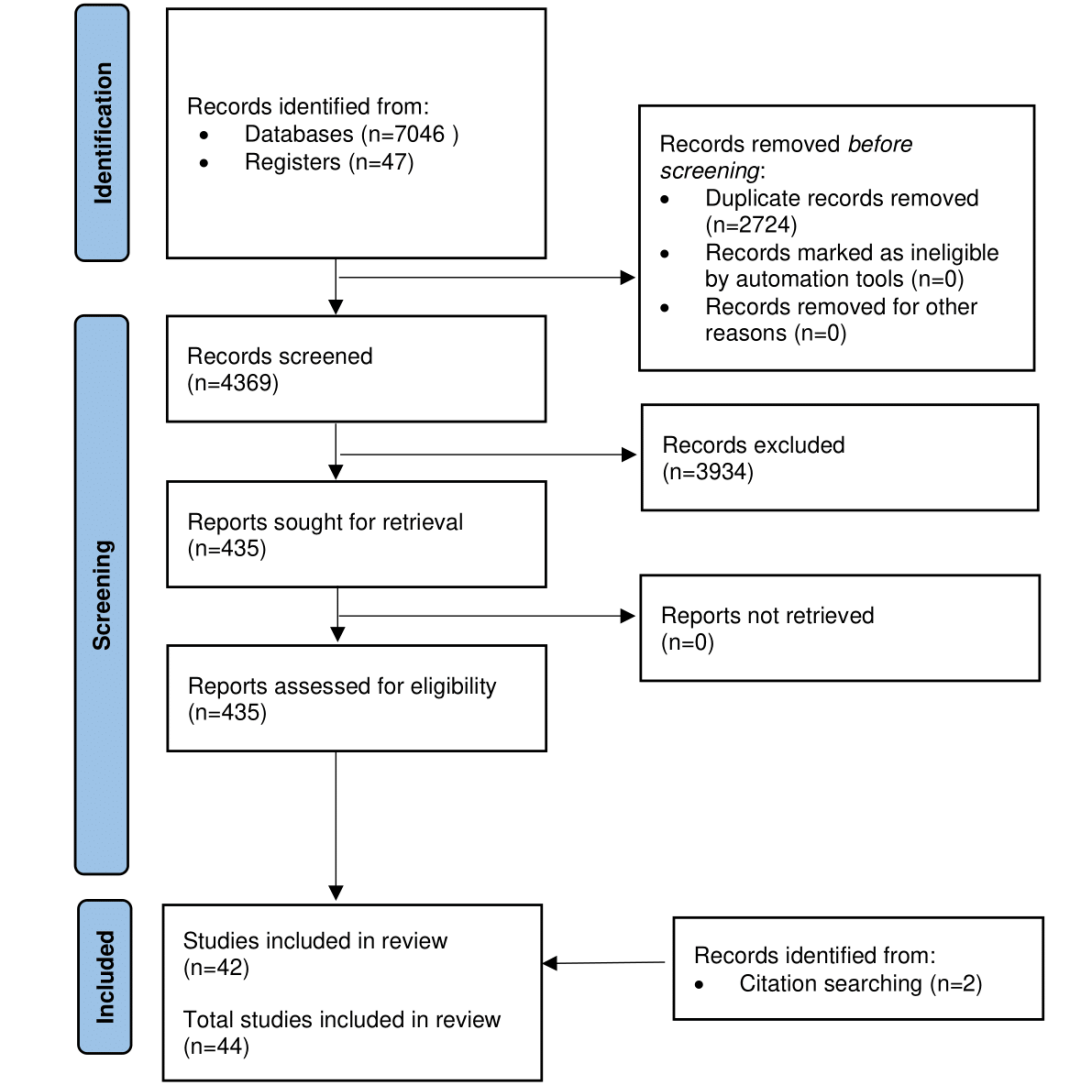
PRISMA_2020_Flow_Diagram.

A total of 7093 records were initially identified. After removing duplicates, 61.6% (4369/7093) of the records were retained for title and abstract screening. A total of 6.13% (435/7093) of the records underwent full-text screening. A total of 44 records examining 50 apps used in dementia care were included in the final review for data extraction (Figure 1 [44]). These same records were included for quality assessment.

### Study Characteristics

Multimedia Appendix 3 [45-88] presents information on the study and app characteristics. Of the 44 included studies, 18 (41%) applied a mixed methods approach, 9 (20%) were qualitative studies, 5 (11%) were case studies, 4 (9%) were quantitative studies, 4 (9%) were RCT studies, and 4 (9%) were non-RCT studies. A total of 12% (6/50) of the apps included in this review were examined in multiple studies [45-50]. Multimedia Appendix 4 [45-88] summarizes the study design and critical appraisal tools.

The included 44 studies were published between 2009 and 2022. There was an upward trend in the number of publications after 2015, with 89% (39/44) of the studies published after 2015. Authors from the United Kingdom contributed the most publications (15/44, 34%), followed by the United States (11/44, 25%) and Malaysia (3/44, 7%) [51-53]. Most studies (36/44, 82%) were conducted in their own countries. Collaboration among countries was rare. Only 18% (8/44) of the studies [48,54-60] involved global collaborations. Most studies (27/44, 61%) reported receiving funding, whereas 36% (16/44) did not disclose their funding status. Only 2% (1/44) of the studies explicitly indicated that they received no financial support [61]. A total of 70% (31/44) of the studies were published as journal papers, and 30% (13/44) were conference full-text proceedings.

### App Characteristics, Including Device Type and OS

A total of 50 apps were included in this review. Most apps (41/50, 82%) were offered in English, whereas 22% (11/50) were multilingual, with 12% (6/50) [54,62-64] including the Chinese language. With respect to the OS, of the 50 apps, 13 (26%) used only the iOS platform, 12 (24%) used Android only, and 15 (30%) were compatible with both Android and iOS. In total, 4% (2/50) of the apps could be used on the Microsoft Windows platform [62,65], and another 4% (2/50) were web-based applications in addition to their native apps [62,66]. A total of 20% (9/44) of the studies did not mention the software platform of the app [53,58,67-73]. Regarding device type, more than half (23/44, 52%) of the included studies used tablet computers only, with the most common tablet being the iPad (12/23, 52% of the studies). A total of 23% (10/44) of the studies used only smartphones, and 9% (4/44) [59,71,74,75] used a combination of both tablets and smartphones.

Of the 35 newly developed apps, only 5 (14%) were commercially published on a web store [60,64,66,76,77]. All these new commercially published apps received financial support except for 1 that did not mention the financial status [64]. The remaining 30% (15/50) of the apps were commercially available apps that were not specifically developed for people living with dementia but were examined as apps used in dementia care in the included studies. These included game apps (10/15, 67%) for leisure activities [54], Our Story [61], Google Calendar [63], the TouchOSC music-making app [78], TomTom [55], and GoTalk NOW [62], which were all tested with people living with dementia. For example, the Our Story app was originally designed to develop children’s reading and social skills [89,90] but was used for reminiscence purposes with people living with dementia in a study [61]. Such apps are valuable to include in this review to demonstrate the variety of tools that may benefit people living with dementia.

### Participant Attributes

Multimedia Appendix 5 [45-88] presents the participant attributes. The study participant types included people living with dementia or their informal caregivers. Half (22/44, 50%) of the studies included both people living with dementia and informal caregivers. A total of 27% (12/44) of the studies recruited only people living with dementia, and 18% (8/44) of the studies [46,66-68,71,75,79,80] involved only caregivers. A total of 5% (2/44) of the studies [76,81] did not specify the participant type, one of which [76] combined participants with cognitive impairment, whereas the other [81] did not specify whether the participants were informal caregivers or people living with dementia. A total of 30% (13/44) of the studies did not report participants’ stages of dementia. Among those studies that reported dementia severity (22/44, 50%), studies that included mild dementia were the most common (18/22, 82%), followed by those that included moderate dementia (10/22, 45%) and early stages of dementia (5/22, 23%). Only 9% (2/22) of the studies involved people living with dementia at the severe stage [54,82]. In total, 18% (8/44) of the studies specified that the participants were diagnosed with AD [52,56,62,63,65,69,83,84].

The number of recruited people living with dementia and caregivers was small across all the studies. Most studies (35/44, 80%) included ≤30 participants of each type (Multimedia Appendix 6). Only 9% (4/44) of the studies had >30 participants living with dementia, with sample sizes of 112 [48], 80 [77], 70 [84], and 54 [54]. In total, 14% (6/44) of the studies with caregiver participants had a sample size >30, specifically samples of 98 [48], 40 [59], 36 [46,79], 35 [70], and 31 [47]. A total of 649 people living with dementia (sample sizes ranging from 1 to 112) and 568 caregivers (sample sizes ranging from 1 to 98) were represented in the final included studies. A total of 2% (1/44) of the studies did not report sample sizes for participants living with dementia [73], and 5% (2/44) of the studies did not report sample sizes for informal caregiver participants [77,84]. Of the 35 studies that included people living with dementia, 9 (26%) did not report age information (ie, did not report mean age or age range). In total, 9% (4/44) of the studies had unclear reporting of age information of people living with dementia [64,70,74,76]. The mean age of people living with dementia was 74.4 (range 39-98) years. Of the 30 studies that recruited informal caregivers, approximately half (14/30, 47%) did not specify any informal caregiver age information. The mean age of the informal caregiver participants was 62 (range 17-91) years. Participants’ ethnicity and socioeconomic status were rarely addressed (16/44, 36%). Over half (23/44, 52%) of the studies collected information about the participants’ previous experience with computer skills or mobile devices.

### Research Aim 1: Types and Features of Apps in Dementia Care for People Living With Dementia or Their Informal Caregivers

#### Overview

A total of 76% (38/50) of the apps were used specifically for people living with dementia, whereas 12% (6/50) were developed solely for caregivers [66,67,74,75,79,80] and 12% (6/50) were developed for both people living with dementia and their informal caregivers [60,68,71,82,84,85]. In total, 60% (30/50) of the apps had a single feature, meaning that an app served a single purpose, such as apps for reminders, reminiscence, or brain games [84]. A total of 40% (20/50) of the apps had multiple features, meaning that different features were brought into 1 app platform, such as an app offering both reminiscence and reminder features [84]. It should be noted that 60% (12/20) of the multiple-feature apps were tested most recently in 2020 and 2021. The following sections will discuss the features of the apps used by people living with dementia followed by those of the apps used by informal caregivers.

#### Apps in Dementia Care Used by People Living With Dementia

Multimedia Appendix 7 provides a summary of the features of the apps for people living with dementia. Among the 32 studies, apps focusing on cognitive stimulation were the most common (n=17, 53%), followed by reminders or prompts for people living with dementia to complete ADLs (n=13, 41%), social support (n=12, 38%), and scheduling for people living with dementia (n=7, 22%).

Among the cognitive stimulation apps, reminiscence was the most common approach, followed by brain games. The most common features of reminiscence included but were not limited to viewing old photos and videos from their past and listening to familiar music [45,50]. Ryan et al [45] found that people living with dementia enjoyed viewing their old photos the most, followed by listening to familiar music and viewing videos. People living with dementia preferred personalized media rather than general media [49]. Similarly, another study found that people living with dementia enjoyed nostalgic songs with which they were familiar. The study also found that participants happily danced to the music [72]. People living with dementia loved interacting with their own old photos, photos of their family and friends, and photos of family holidays they spent together [45,49,59].

Reminder apps included reminders for taking medication [57-59,81,84], appointments (ie, meeting with family physicians or friends) [57,63,65,68,84], daily activities (ie, watch the favorite TV show) [52,59,86], mealtimes [59,81], special events (ie, birthdays) [56,84], religious events (ie, prayer time and church visits) [59,84], doing chores [84], and weather reminders (ie, warning of rain and snow) [59]. In total, 7% (3/44) of the studies focused on apps providing step-by-step prompts for people living with dementia to complete daily tasks, such as making a phone call or a cup of coffee [69,76,86].

There were 6 kinds of social support offered by the apps. First, *connection* referred to the app’s ability to give people living with dementia quick access to the contact information of their family or friends and other essential people (eg, family physician). A photo or the relationship between the contact person and the user would be displayed to help people living with dementia quickly identify them. Second, *recognition* meant that the app helped people living with dementia identify a person, face, or object. Third, *calling for help* was a feature that allowed people living with dementia to call a predefined contact person (ie, usually informal caregivers) if help was needed. Fourth, *needs support* referred to specific assistance for people living with dementia to meet their daily needs with the help of their caregivers [59]. For example, a task message would be sent to the predefined informal caregivers regarding the request of people living with dementia using the app [59]. Fifth, *communication aids* meant that the app offered an augmentative and alternative communication method to help improve the communication of people living with dementia [62]. Finally, *location-based review* referred to the app’s ability to allow users to provide a review and rating of a location they visited [85].

#### Apps in Dementia Care Used by Informal Caregivers

Multimedia Appendix 8 identifies and describes the app features used in dementia care that targeted informal caregivers as the end users. The most common features of the apps included activities that enabled caregivers to engage the people living with dementia in an activity or task (eg, playing a game together) [60,67,75,82] and providing caregiving support [66,67,75,79].

### Research Aim 2: Privacy and Safety Features of the Apps From the Perspective of Both End Users and Developers

Only 2% (1/44) of the studies mentioned safety and security concerns about the app from the users’ perspective during their usability study [60]. This study involved people living with dementia, their informal caregivers, and home care workers. One home care worker raised privacy and safety concerns during an interview:

For me the negative side is...because the information can be accessed easily by everybody, we have difficulties in differentiating between true information and hoax.

60

With respect to personal information, apps from 59% (26/44) of the studies required explicit entry of personal information, defined as any information that could identify a person, such as a person’s name, date of birth, home address, photos, or voice [91].

In total, 9% (4/44) of the studies discussed the various security features used in the apps [66,71,74,82]. One study applied a secure certificate (Secure Sockets Layer) to the app to ensure encrypted communication between users and the server [66]. Passwords were required to access the data, and the data were encrypted and stored in a database. No data were saved after web browsing. It was indicated that the participants’ health information would not be transferred to an external server or reserved for future research purposes [66].

In the study by Reyes et al [71], user data were stored on an Amazon Elastic Compute Cloud. The authors argued that Amazon services were chosen because of their ability to provide data backup support, security features for data, and advantages in data analytics [71]. In another study [74], an Advanced Encryption Standard was applied to encrypt the data that were collected and stored on the MySQL database and Amazon Simple Storage Server Bucket (ie, cloud storage) [92]. The users’ media were stored on the Amazon Simple Storage Server Bucket, and the users’ information was stored on the MySQL database [74]. Schultz et al [82] stated that the data were managed and stored securely in a back-end system. However, none of these 4 studies mentioned whether the data could be deleted (by users or others) or the length of time that the data would be stored. In addition, 5% (2/44) of the studies used a database to store and retrieve the data [79,81]. Notably, there was no information on whether security features were applied to the database. In another study, participants were asked to use pseudonyms, and their locations were partially anonymized [61]. However, none of the studies mentioned the length of time that the data would be stored or whether users could access and delete the data themselves.

Across these 26 apps that required users to enter their personal information, 8 different privacy policies were found [55,61-64,66,76,77]. Our analysis of the privacy policies identified 17 themes (ie, commonalities). Multimedia Appendix 9 shows the privacy policy themes and their descriptions. It is important to note that not all the themes were reflected in all the privacy policies; the only commonality found in all the privacy policies was the *contact information* and *data entered by end-users* themes [93-100]. The other frequently occurring themes were privacy policy changes [95-100], app owner (ie, the app developer who creates an app) shares end users’ data [93,95-98,100], data retention [93,95-98,100], security features [93,96-100], data ownership [93-95,97,98,100], and data use [93-97,100]. Less common aspects of privacy policy themes included age restrictions for users [95,96,100], procedure for misuse of data [95,96,98], data transfer [95,97], and jurisdictional data protection laws such as the General Data Protection Regulation [95,98].

The privacy policies varied with respect to what information was included and the level of detail provided. The Memory Matters policy encompassed the most content, with 15 privacy policy themes [95], followed by the privacy policies of Google Calendar and MapHabit, which covered 14 themes [97,100]. The GoTalk NOW privacy policy covered the least themes (Multimedia Appendix 10). In addition, several aspects of the privacy policies were not detailed. For example, in the security features theme, the GoTalk NOW privacy policy did not specify security methods such as data encryption during transit or data storage via a secure server. The policy only stated that the app owner (ie, app developer) followed the laws and regulations to protect the users’ personal information and data security [99]. A total of 38% (3/8) of the policies also had generally broad statements such as “personal data would be collected” [94,95,98].

Several issues were identified in our analysis of the app privacy policies. For example, poor readability (eg, several sentences were incomplete in the Memory Matters privacy policy [95]), 50% (4/8) of the policies not having “privacy policy” as the title [93-95,98], and poor accessibility (eg, 1 policy’s link was no longer active [100]). Web links were also found in place of the privacy policies; however, upon further examination, these links led to terms of service rather than privacy policies [98], the privacy policy of the university that developed the app and was not specific to the app [94]. Overall, the privacy policies lacked consistency in content and varied widely with respect to detail and quality.

### Research Aim 3: Methodologies Used for the Usability Evaluation of Apps Used in Dementia Care

In total, 5 methods were identified for usability evaluation: questionnaire, interview, observation, logging, and focus group. Questionnaires were the most adopted approach (28/44, 64% of the studies), followed by interviews (25/44, 57% of the studies), observations (25/44, 57% of the studies), logging (5/44, 11% of the studies), and focus groups (5/44, 11% of the studies). Most studies (29/44, 66%) applied more than one method to assess usability: 36% (16/44) applied 2 methods, 25% (11/44) applied 3 methods, and 5% (2/44) used 4 methods to evaluate usability [47,52].

Of the 28 studies that used questionnaires, 17 (61%) used self-developed questionnaires. The questionnaires asked about the utility, usability, usefulness, functionality, learnability, satisfaction, ease of use, effectiveness, efficiency, and acceptability of the app and participants’ experience, opinions, perceived interest, motivation to use the app, and suggestions for improvement of the app. In total, 32% (9/28) of the studies used modified questionnaires [47,48,53,56,60,66,75,83,84]. A total of 21% (6/28) of the studies used standard questionnaires [57,64,67,68,75,86], and the SUS was used most often [57,64,68,75,86]. The other standard questionnaires used in the studies were the Single Ease Question and Mobile Phone Usability Questionnaire [64,67]. Qualitative methods were also used in the form of audio [45,47,55,59,69,74,75,80,87], field notes [45,47,69,80], or video-recorded [62] interviews and focus groups [47,60,67,75]. In addition, several methods were used for the observational approach, which included *think aloud* [64,71,78], field notes, and audio [50,55,57,64] or video recordings [55,62,64,67,72,82]. Finally, logging is an objective way to record a person’s actual app use [64]. For instance, 11% (5/44) of the studies used log data—a file generated by an app itself in real time with user-app interaction information [48,49,52,56,66]. Log data included the frequency and duration of app use, steps taken and errors made in completing a task, prompt frequency or guidance required by people living with dementia to complete a task using an app, and task completion rate and completion time. The frequency of app use is defined as how often an app is used. The duration of app use is the amount of time a user uses an app. The task completion rate is the number of times a task can be completed. Task completion time is the time required by a user to complete a given task using the app [64]. Prompt frequency or guidance is the number of times a user requires reminders or prompts or guidance to complete a task [50,57,59].

### Research Aim 4: Results of the Usability Evaluation of Apps Used in Dementia Care

#### Overview

Most studies (36/44, 82%) showed that participants had a positive experience interacting with the app. Participants from 7% (3/44) of the studies, which assessed a cognitive stimulation game app [88], a facial recognition app [70], and an app to help people living with dementia with ADLs [58], explicitly expressed that these apps were *not useful* and showed *low interest* in using the apps in dementia care [58,70,88]. The cognitive stimulation game app was described as unsuitable for people living with dementia because of its complexity [88] and the fact that people living with dementia would constantly require help from a third person to use the app successfully [88]. People living with dementia reported that they did not think the facial recognition app was useful and that it would not affect their social interactions or QOL. Thus, people living with dementia did not want to continue using the app after study completion [70]. In the study by Lai et al [58], the authors developed an app with 4 features: simplified phone calls or SMS text messages to friends or family members, reminders, 1-click emergency calls, and a simplified navigation system for people living with dementia. The results showed that people living with dementia had low interest in the app even after a demonstration. People living with dementia were against the idea of having too much interference from the caregivers in their life. For example, participants living with dementia did not like caregivers updating their appointments remotely or being able to view or monitor their location [58].

Only 2% (1/44) of the studies mentioned accessibility and the barrier to internet access for people living with dementia in rural settings compared with urban settings [60]. A total of 9 themes were identified from app usability testing, which are described in the following sections.

#### App User Interface

The studies highlighted that apps should be intuitive to use [46,65,71,80], which could enhance the learnability of people living with dementia [65] and facilitate independent app use [61]. The text and icons should be large enough to improve readability for people living with dementia and older adults, especially individuals with vision problems [50,52,61,71,88] or limited dexterity, which reduces their ability to tap or hold the icon or button [61,88]. Please see Table 1 for more detail.

**Table 1:** Summary of suggested app user interfaces.

	Suggestions	Study
Text	Text size should be large to improve readability, especially for people living with dementia with vision impairments. Decrease text density by minimizing the use of text and using icons or pictures where applicable instead.	Rai et al [47] Boyd et al [50] Hashim-de Vries et al [52] Mohd Hassan et al [53] Quintana et al [57] Chaudhry and Smith [68] Reyes et al [71] Rettinger et al [75] Hackett et al [86] Boyd et al [88]
Icons	Use larger icons. Icon names should be intuitive and familiar.	Boyd et al [50] Mohd Hassan et al [53] Quintana et al [57] Critten and Kucirkova [61]
Colors	Colors should have a stark contrast. Avoid using light colors, which make it harder to read.	Boyd et al [50] Reyes et al [71] Yamagata et al [73]
Illustrations	Illustrations can help people living with dementia understand the app components more easily.	Reyes et al [71]
Visual stimuli	Visual stimuli such as animation can be used to attract users’ attention.	Boyd et al [50] Rai et al [60] Wu et al [72]
Voice control	A voice feature could be added to an app for users who do not want to type or read on the app.	Mohd Hassan et al [53] Asghar et al [59] Hashim-de Vries et al [52]
Image background	Backgrounds should use a brighter color to make a strong contrast or should be plain with a single color.	Mohd Hassan et al [53]
Language use	Plain language should be used; text should avoid jargon. Avoid using stigmatizing words such as demented people. Avoid using homonyms such as album.	Brown and Kim [67] Chaudhry and Smith [68] Boyd et al [50]
Buttons	Size of the buttons should be large to make them easily visible. Layout should be such that there is ample spacing between buttons to prevent errors. The button should have dimensions, for example, a drop shadow or animation, to indicate to users that the button can be clicked or selected.	Hackett et al [86] Imbeault et al [65] Yamagata et al [73] Mohd Hassan et al [53]
Pop-up advertisements	Pop-up advertisements should be avoided.	Groenewoud et al [54]

#### Physical Considerations

People living with dementia may face challenges when using mobile devices because of their vision [52,60,61,71] or hearing impairments [47], loss of touch sensation, motor impairments (ie, hand tremors or spasms), or physical pain [47,78]. One study indicated that physical pain could reduce the time required to use an app or serve as a distraction from the pain [47]. Issues such as glare could make it even worse for people living with dementia [73,87], so it was recommended to use a screen protector to reduce glare [73]. Some people living with dementia may not be able to hold the device while interacting with it because of its size and weight [61,73] and their reduced dexterity [61,88]. One study reported that people living with dementia might find it hard to use the keyboard to type because of poor hand coordination or swollen fingers [57,61]. People living with dementia may accidentally press unintended screen items, or their finger may rest on the screen by accident to better hold the device [88]. As such, a stylus or tablet pen could ease the dexterity issue and reduce the screen’s unintended touch [57,88].

#### Screen Size

Owing to vision problems, people living with dementia may prefer a bigger screen [52,73]. However, there was no consensus regarding the size of the tablets for people living with dementia. One study concluded that the screen should not be smaller than 10.2 inches [73]. Although some people living with dementia preferred a bigger screen, others thought the smaller size, such as a 7-inch touch screen, would be more portable [47,52,57,73,74,88].

#### App Interaction Challenges

The studies reported that people living with dementia may be unable to choose the correct widget or app icon [65,101] and may not understand how to swipe, tap, or drag icons to navigate various apps [54,101]. For example, people living with dementia may use the top of the fingernail to swipe instead of a fingertip. It was found that older adults, especially people living with dementia, were not sensitive to the difference between using fingernails and fingertips to respond [73]. In addition, people living with dementia may press a widget or home screen for too long and accidentally delete or edit the home screen [57,101]. Instead of tapping the screen, some people living with dementia may tend to drag their fingers across the screen [73]. People living with dementia may find it challenging to touch the interface accurately and accidentally press the taskbar instead of the app button [65,73].

#### Apps Should Fit User Needs

##### Introduce an App at the Right Time

Timing with respect to dementia progression and the abilities of people living with dementia is an important variable for app usability. One study suggested that an app that could track the symptoms of people living with dementia may not be useful for caregivers of people living with dementia at a later disease stage as there may not be notable symptom changes [46]. In the same way, individuals with severe dementia may require a significant amount of help from a third person [45,48,69,88]. One study flagged how informal caregivers could end up persuading their loved ones to use the app against their wishes, thereby causing tension in their relationship [69]. In contrast, if an app is introduced too early in the progression of dementia, users may not be interested in using it. For example, high-functioning people living with dementia may not be interested in a cognitive game app developed for people living with dementia as they may not find it challenging [47,54].

##### Personalization of an App

Caregivers also indicated the importance of app flexibility in meeting their various needs [46,80]. The customization or personalization of an app is desired to suit a range of users. For example, not all caregivers care for people with AD. Therefore, if the resources or education included in caregiver support are only AD-related, informal caregivers of people with other types of dementia will find little utility in the app [46].

#### Limited Insight of People Living With Dementia Into Their Own Needs Regarding the App

One usability study indicated that there may be a reported discrepancy between people living with dementia and their informal caregivers [58], specifically that people living with dementia may not realize how often they forget appointments or become disoriented outdoors. In contrast, informal caregivers who provide daily care may be more cognizant of these incidents. For instance, people living with dementia may not believe that apps with navigation or reminder features are useful or relevant to them as much as their caregivers do [58].

#### Stigma

A new app related to dementia care could make people living with dementia feel stigmatized. For example, an app makes people living with dementia feel incompetent in remembering or performing their daily tasks, which could result in unwillingness to use the app [29,54,58,69]. One study revealed that people living with dementia could have a negative experience with a game app because of the fear that their cognitive impairment will be exposed [54]. A study on using a prompt app to remind people living with dementia to complete daily tasks also suggested that people living with dementia might feel embarrassed using the app as high use levels may highlight their memory loss [69].

#### Lack of Technological Experience

Despite the ubiquity of smart devices nowadays, many older adults still have limited experience with mobile technology [60,61,66]. Lack of experience with technology could result in fear of using it [57,60,62,66]. The study by Ekström et al [62] found that users were new to operating a tablet with an app to aid communication. One participant living with dementia was unfamiliar with the tablet, making them more insecure when using the device, so they were more passive during a conversation [62]. Similar results were found in another study, where participants unfamiliar with tablets were more insecure about using the tablet and app [57]. One study concluded that an 11-week study period might not be sufficient for users to get used to a new app [66].

In contrast, users are more likely to engage with an app if they have more experience with mobile technologies [49,63,69]. Users are more confident in learning to use the app and more likely to use it daily [47,49,57,69]. One study found that users with more technological experience rated app usability higher [75]. Another study showed that a participant living with dementia had a positive experience using a reminder app because of her familiarity with smartphones [63].

#### Technical Support

As mentioned previously, technological experience can play an important role in app use. Users need to receive training or instructions to understand apps and mobile devices [50,61]. Training or instructions will help build confidence so users will feel more comfortable using the app [61]. The studies indicated that people living with dementia would need the training to use an app and prefer a demonstration to show them how to use the app and mobile device rather than explore the app and device themselves [57,63,86]. One of the studies pointed out that the importance of a proper introduction to the app could motivate the participants’ independent use, especially for those with less experience with the technology. It also emphasized modifying the instruction level according to the user’s technology experience [57]. The users also appreciated the paper-based manual of the app to help them remember how to use it [57,80].

In addition, support from users’ own networks, such as friends and family, is important [47,60,69]. It was found that both people living with dementia and their informal caregivers would need support during technology use, which is especially true for people living with dementia [60]. People living with dementia often turn to their children or grandchildren for assistance as they are relatively more technologically savvy [60]. People living with dementia may prefer that family members help set up the app to use it successfully [70]. Some people living with dementia may find it useful to connect with other users of the same app for technological issues [47,60]. As users may constantly have questions about a new app or device, it would be crucial to have continuous support, such as from a written manual or a contact person, when a question is raised [80,82]. Ongoing support would also help with app retention [82].

## Discussion

### Principal Findings

#### Overview

To the best of our knowledge, this is the first systematic review to examine the landscape of available apps in dementia care for both people living with dementia and their informal caregivers in a home environment. Notably, our review identified and analyzed various app features, highlighting the most investigated aspects for people living with dementia and their informal caregivers. We also extensively discussed and addressed privacy and safety concerns in app development, including an analysis of app privacy policies. This review offers valuable guidance for selecting appropriate methodologies when conducting usability studies with people living with dementia and their informal caregivers. Finally, we provided a comprehensive overview of app usability outcomes, providing a holistic view of our findings.

#### Research Aim 1: Types and Features of Apps in Dementia Care for People Living With Dementia or Their Informal Caregivers

The 3 most studied app features for people living with dementia were cognitive stimulation, reminders or prompts to complete ADLs, and social support. As stated in other studies, these activities are considered meaningful for people living with dementia and could potentially improve their QOL [102-104]. Cognitive stimulation is a nonpharmacological intervention that provides various activities to stimulate cognition, such as memory, logical judgment, attention, and language skills [105,106]. It can improve brain function and prevent its degradation [72].

It has been shown that reminiscence greatly affects the cognitive wellness of people living with dementia and improves their QOL [45,107]. However, precautions are needed when conducting reminiscence. It was observed that photos with many people were not recommended as people living with dementia may not be able to identify the faces [52]. In addition, extra attention should be paid when selecting materials for reminiscence to ensure that the materials elicit happy memories and not sad or painful ones [45,61] for the people living with dementia or their informal caregivers [45,108]. For example, photos that remind them of the loss of a family member could be significant for caregivers but not for people living with dementia [108].

Reminders of daily activities could help people living with dementia remain independent [104]. However, not all reminders are preferred by people living with dementia, such as activities that can be performed with their family or friends (eg, taking a walk or checking their blood pressure [59]). As such, an app developed to replace caregivers would not be favored by people living with dementia. Users found the reminder feature useful for daily tasks, including medication times and dosages, religious activities, mealtimes, and appointments [58,59,68,86].

Many people living with dementia live at home [15] with their informal caregivers [8], who greatly influence app adoption for people living with dementia [48,57]. People living with dementia would be more likely to accept an app when their informal caregivers are involved [48,77], and people living with dementia are more engaged when using an app in their caregivers’ presence [77]. The use of apps could allow both people living with dementia and their informal caregivers to spend more time together and increase their feelings of connectedness [47]. For example, apps that prompt conversations and effective communication could enable meaningful dialogue and improve the dyadic relationship and quality time [47]. The development of apps for both people living with dementia and their informal caregivers could have benefits for both types of end users individually as well as strengthening their relationship.

Other studies have shown that receiving support from a caregiving network (ie, help from friends or other family members) could have a beneficial impact on maintaining the QOL of people living with dementia and their informal caregivers [109-111]. Oftentimes, secondary caregivers are involved in care [46,112]. These caregivers could act as mediators to share different care tasks and support [46,109]. Thus, an app with a feature connecting family members or friends involved in care could be useful in the care management of people living with dementia [46].

In addition, special attention should be paid to the app instruction or guide (ie, *Help* button), personalization (ie, *Edit*), and rewards features. Although these features were not mentioned in most studies, they are considered incentives and could boost app engagement [77,80,82,86]. It was found that users would greatly appreciate the additional explanation of an app. A *Help* button with tips or instructions was suggested [47,68,80]. The instructions should be written using lay language to increase accessibility for people living with dementia. Ideally, instructions should be able to adapt to the user’s computer experience [57]. An app should be customizable to meet users’ needs [46,80]. An *Edit* feature could be added to the app to allow users to change the setting, such as the font size, background, music, and information sharing [57,68,76]. Finally, both people living with dementia and informal caregivers found reward systems beneficial, and these could help motivate their ongoing use of the app [77,80,86].

The results of this review indicate that 60% (30/50) of researched apps were single-feature apps (ie, served 1 purpose), which meant that users needed to download multiple apps to meet their needs [84]. Another limitation is that most apps (39/50, 78%) offered only 1 language. These limitations could significantly restrict the number of users. To make an app more widely used, we recommend having multiple features and offering several languages.

#### Research Aim 2: Privacy and Safety Features of the Apps

The literature indicates that people living with dementia are concerned about their privacy and security [113,114]. However, this review only identified 1 study that discussed the safety and security concerns of users from the perspective of a home care worker [60]. This suggests that more research is required to examine the perspectives of people living with dementia and their informal caregivers on privacy and security related to mobile apps. Our findings also indicated that privacy and security were not a priority for developers, organizations, and researchers when developing apps based on the few studies that described these privacy features (4/44, 9%). Our findings align with those of previous studies that suggested that developers lack awareness and knowledge when applying security and privacy measures during app production [115,116]. None of the studies described the length of time during which data were stored or whether users could delete their data, which raises critical ethical questions regarding data ownership and private interests related to people living with dementia. This review also revealed that the privacy policies for commercially available apps are inconsistent and lack detail, with important content missing according to the General Data Protection Regulation [117,118].

None of the usability questionnaires used in the studies included security- and privacy-related questions despite 61% (17/28) of the questionnaires being self-developed by the researchers rather than standardized and validated tools. Despite the importance of privacy in technology adoption [16,27,28] and the potential algorithmic bias or harm toward older people (eg, digital ageism [119]), our findings suggest that developers and their organizations have not seriously addressed or investigated the issues related to privacy [120,121]. More research is required to investigate mobile device–related privacy and security, especially as they relate to people living with dementia, as the current research in this area is still in its nascent stages.

Although there are publicly available resources to build privacy and security tools, such as checklists through app stores or computing platforms [116], our findings can be used to further advance a consensus about privacy policies. In particular, the 17 themes from research aim 2 could serve as a general framework to assist developers, organizations, and researchers in creating detailed and concise privacy policies. In addition, we urge organizations to protect users’ data [122,123] proactively, for example, ensuring that a transparent, concise, and valid privacy policy is made available for users and that all web links are active and updated. Finally, more public education about privacy and security risks related to mobile apps can enhance the vigilance of formal and informal caregivers and people living with dementia when choosing or using an app [122,123].

#### Research Aim 3: Describe the Methodologies That Have Been Used for Usability Evaluation

This review found 5 methods to evaluate usability: observations, interviews, focus groups, logging, and questionnaires. Techniques for these methodologies include think aloud, video and audio recordings, and field notes [64,124]. The most commonly used method in our review were questionnaires administered at the end of the intervention. The most commonly used standard questionnaire was the SUS; however, studies found that people living with dementia may not be able to complete the SUS because of its double-negative questions [57,64,86]. Usability questionnaires may not assess the true performance of people living with dementia as questionnaire completion usually occurs after the intervention, which requires recalling the user experience [64,86]. One study compared SUS results between caregivers and people living with dementia [64]. It showed that people living with dementia provided higher ratings on the SUS, meaning that the app was more usable for people living with dementia than for caregivers. However, caregivers’ higher task completion rates suggested that the app was more usable for caregivers [64]. Questions have been raised regarding whether the results of questionnaires are reliable and valid when used to collect complex feedback from people living with dementia [57,64,86]. Similarly, the think-aloud technique was used in several studies; however, the literature indicates that this methodology may be unsuitable for people living with dementia [50,64,71]. The think-aloud process requires users to express their opinions without analyzing their thoughts while performing specified tasks [125]. This method could overload the working memory of people living with dementia and lead them to provide inaccurate information [50,64]. Although the results will depend on the severity of dementia, the aforementioned methods are more prone to errors as they are based on self-report and fallible to recall bias, which would result in inaccurate usability testing [126]. For instance, people living with dementia may provide an answer that they think others would give [126]. Therefore, thoughtful consideration of the methodologies used to engage with people living with dementia is required, and caution may be needed when interpreting the results of unvalidated posttest questionnaires with people living with dementia.

In contrast, observation and logging provide more objective measures. Observation requires researchers to document detailed information about what they see when users interact with an app. The duration and frequency of app use and task completion rate and time could be recorded during observation. To make the results from observation more reliable, in addition to the presence of the observers taking notes, video recording could capture users’ reactions and their words when interacting with an app and then be assessed by 2 researchers independently [64]. Logging was another form of objective data collection, which is the user data generated from the app with detailed user-app interactions (eg, frequency of app or app feature use, duration of app use, and task completion time).

In summary, applying multiple usability evaluation methods is recommended [124]. Combining objective and subjective measures is suggested to better understand users’ needs [47,49,101]. Objective measures are meant to improve the validity and reliability of the usability results. Although logging could be more promising and cost-effective than observation methods as it reduces human involvement in recording and analyzing user-app interactions [126,127], it is also important to consider the methodology that would achieve the most relevant results in the given context. By assessing the suitability of different usability evaluation methods and considering their alignment with the specific research goals, we can ensure that the selected approach not only reduces bias and enhances efficiency but also provides the most meaningful and contextually relevant outcomes. When enrolling people living with dementia to complete questionnaires, precautions should be taken: questionnaires should be understandable, and long questionnaires with jargon and double negatives should be avoided. Some questionnaires, such as the SUS, may not be suitable for people living with dementia [57,64,86].

#### Research Aim 4: Summarize the Results Obtained From the Usability Evaluation of the Apps

Our review showed that most studies (36/44, 82%) reported a positive experience of interacting with the apps. A usable and intuitive-use app could increase user engagement [45], help reduce users’ cognitive overload, and build their confidence in using it. In contrast, an unintuitive app could reduce interest in use [69]. A lack of intuitive navigation could also hinder app adoption [58,69]. Our results under research aim 4 could guide researchers or developers in creating new apps to enhance usability for people living with dementia. This body of evidence shows that, although apps used in dementia care are a usable digital intervention that can provide a variety of supports for people living with dementia and their informal caregivers, our results highlight several important design and privacy concerns that should be accounted for in future research.

### Strengths and Limitations

This systematic review makes significant novel contributions as the first review that systematically summarizes the features of apps for people living with dementia and their informal caregivers in dementia care, as well as the security and privacy features of apps. There are several strengths to this systematic review. A broad database search was conducted with the help of an information specialist. This review used predefined inclusion and exclusion criteria for the patient population and interventions and 2 reviewers to ensure an appropriate and robust approach. However, there are also some limitations to this review. Only 1 reviewer conducted the full-text screening. This review only included studies in the English language, which may mean that empirical studies from other non–English-speaking countries were not included. This review only covered apps that underwent usability testing and did not include all commercial apps that did not undergo empirical usability testing.

### Conclusions

People living with dementia and their informal caregivers can benefit from using mobile technology to facilitate care. Apps in dementia care are promising tools for improving the QOL of people living with dementia and their informal caregivers. This review highlights the landscape of existing researched mobile apps for people living with dementia and their informal caregivers in dementia care. To advance future work, our findings provide a preliminary framework that can direct app developers or researchers regarding privacy policy generation and provide guidelines for app developers or researchers producing future apps in dementia care and recommendations for future researchers on how to better conduct usability studies. This review also draws attention to the lack of transparency regarding privacy and security when using these apps. Our results highlight the need for more development and research to address these ethical concerns.

## Appendix

Multimedia Appendix 1 PRISMA (Preferred Reporting Items for Systematic Reviews and Meta-Analyses) checklist.

Multimedia Appendix 2 Search strategy.

Multimedia Appendix 3 A description of study and app characteristics.

Multimedia Appendix 4 Results of the appraisal of the included studies.

Multimedia Appendix 5 A description of participant characteristics.

Multimedia Appendix 6 The sample size of participants distributed across the studies.

Multimedia Appendix 7 A summary of app features for people living with dementia.

Multimedia Appendix 8 A summary of app features for caregivers.

Multimedia Appendix 9 A description of privacy policy themes.

Multimedia Appendix 10 Tally of themes of each app privacy policy.

## Abbreviations

AD: Alzheimer disease
ADL: activity of daily living
OS: operating system
QOL: quality of life
RCT: randomized controlled trial
SUS: System Usability Scale
