# Supplementary material for: Researched Apps Used in Dementia Care for People Living With Dementia and Their Informal Caregivers: Systematic Review on App Features, Security, and Usability
Source: J Med Internet Res. 2023 Oct 12;25:e46188. doi: 10.2196/46188 (PMC10603562; doi:10.2196/46188)
Supplement: Multimedia Appendix 2 [file jmir_v25i1e46188_app2.doc]

**UPDATE2 Search Strategy and Supporting Documentation
Mobile Apps and Dementia

Completed by: Jessica Babineau MLIS**

**SEARCH DETAILS**

Searches were conducted in Cochrane Central Register of Controlled Trials (CENTRAL, in Ovid), Embase (Ovid), MEDLINE ALL (including Epub Ahead of Print, In-Process & Other Non-Indexed Citations, MEDLINE(R) Daily, in Ovid), APA PsycInfo (Ovid), Dissertations and These Global (Proquest), Scopus, Compendex (Engineering Village), Inspec (Engineering Village), and the ACM Digital Library.

Searches were conducted in May 2018, and were subsequently updated in December 2019 and again in February 2022.

**2019** changes to the databases searches:

- Added “kw” field to textword lines in Embase strategy.
- The “kf” code was corrected to “kw” in CENTRAL strategy

**2022** changes to the database searches:

- Changed “kw” to “kf” in Medline ALL(Ovid) strategy
- Added ez and da to update codes in Medline ALL (Ovid) as per: <https://libraryguides.mcgill.ca/knowledge-syntheses/updating>
- Altered CENTRAL language limits to identify English publications that are not tagged as English
- Updated human limit to Embase as per: <https://utas.libguides.com/SystematicReviews/SearchLimits>
- Renamed PsycINFO to APA PsycInfo (and added to list above - was missing)
- Scopus update code used as per <https://libraryguides.mcgill.ca/knowledge-syntheses/updating>

Search strategies included the use of textwords and subject headings (e.g. MeSH, Emtree) related to (1) dementia or alzheimer’s and (2) mobile apps. Searches were limited to human studies, and English language papers when possible. Searches were developed and conducted by an Information Specialist (JB).

**UPDATED PRE-DUPLICATE REMOVAL RESULTS**

When possible, update limits were used in the database to only import references added since December 2019. Limits used were determined by the following: <https://libraryguides.mcgill.ca/knowledge-syntheses/updating>. The flagged databases below (*) have no working update limits – for these databases, I imported ALL citations, de-duplicated the original and new results within Endnote, and kept the new, non-duplicate records.

*TOTAL Results: 7,093 citations (2,461imporated in Endnote (update 2))*

- * ACM Digital Library: 665 results (Kept 195)
- *Cochrane Central Register of Controlled Trials (CENTRAL): 100 results (Kept 47)
- *Compendex: 1,314 (Kept 363 )
- Embase: 1575 results (Imported 632)
- *Inspec: 834 results (Kept 254)
- MEDLINE: 786 results (362 Imported)
- *Proquest Dissertations and These Global: 59 results (Kept 21)
- APA PsycInfo: 351 results (101 Imported)
- Scopus: 1409 results (486 Imported )

**Note that there remains duplicates between databases, and there may be some overlap between original and new results. A de-duplication screen is required within new results, and comparing new to original results.**


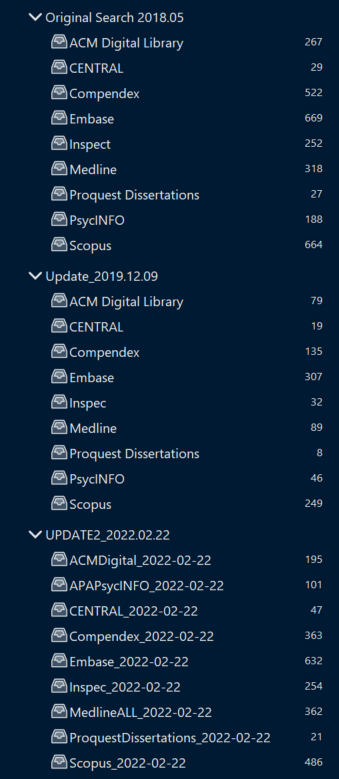


**SEARCH STRATEGIES - UPDATE 2 - 2022-02-22**

Database: **Ovid MEDLINE(R) ALL <1946 to February 21, 2022>**

Search Strategy:

--------------------------------------------------------------------------------

1 exp Dementia/ (187205)

2 Wernicke Encephalopathy/ (1825)

3 Delirium, Dementia, Amnestic, Cognitive Disorders/ (9583)

4 dement*.tw,kf. (132663)

5 alzheimer*.tw,kf. (169210)

6 (lewy* adj2 bod*).tw,kf. (10710)

7 (chronic adj2 cerebrovascular).tw,kf. (737)

8 ('organic brain disease' or 'organic brain syndrome').tw,kf. (800)

9 ('normal pressure hydrocephalus' and 'shunt*').tw,kf. (1444)

10 'benign senescent forgetfulness'.tw,kf. (18)

11 (cerebr* adj2 deteriorat*).tw,kf. (248)

12 (cerebral* adj2 insufficient*).tw,kf. (85)

13 (pick* adj2 disease).tw,kf. (3496)

14 (creutzfeldt or jcd or cjd).tw,kf. (7401)

15 huntington*.tw,kf. (19617)

16 binswanger*.tw,kf. (596)

17 korsako*.tw,kf. (1684)

18 1 or 2 or 3 or 4 or 5 or 6 or 7 or 8 or 9 or 10 or 11 or 12 or 13 or 14 or 15 or 16 or 17 (316453)

19 Mobile Applications/ (9537)

20 exp Computers, Handheld/ (11164)

21 Smartphone/ (7371)

22 Cell Phone/ (9493)

23 Telemedicine/ (32401)

24 (mobile adj3 (application? or app? or device? or technolog* or computer*)).tw,kf. (18512)

25 (portable adj3 (application? or app? or device? or technolog* or computer?)).tw,kf. (6363)

26 (mhealth or m-health).tw,kf. (7819)

27 ((app? or application? or device? or technolog* or platform* or computer program* or software?) adj3 (smartphone? or phone? or cellphone? or tablet? or handheld or hand-held or iphone? or ipad? or android? or touchscreen? or touch-screen?)).tw,kf. (16319)

28 ((app? or application?) adj3 computer?).tw,kf. (4151)

29 or/19-28 (83484)

30 18 and 29 (821)

31 limit 30 to english language (797)

32 31 not (exp animals/ not exp humans/) (786)

33 (201912* or 202*).dt,ez,da. (4078100)

34 32 and 33 (362)

***************************

Database: **EBM Reviews - Cochrane Central Register of Controlled Trials <January 2022>**

Search Strategy:

--------------------------------------------------------------------------------

1 exp Dementia/ (6445)

2 Wernicke Encephalopathy/ (4)

3 Delirium, Dementia, Amnestic, Cognitive Disorders/ (0)

4 dement*.tw,kw. (15064)

5 alzheimer*.tw,kw. (12629)

6 (lewy* adj2 bod*).tw,kw. (490)

7 (chronic adj2 cerebrovascular).tw,kw. (670)

8 ('organic brain disease' or 'organic brain syndrome').tw,kw. (149)

9 ('normal pressure hydrocephalus' and 'shunt*').tw,kw. (91)

10 'benign senescent forgetfulness'.tw,kw. (2)

11 (cerebr* adj2 deteriorat*).tw,kw. (35)

12 (cerebral* adj2 insufficient*).tw,kw. (6)

13 (pick* adj2 disease).tw,kw. (45)

14 (creutzfeldt or jcd or cjd).tw,kw. (74)

15 huntington*.tw,kw. (772)

16 binswanger*.tw,kw. (6)

17 korsako*.tw,kw. (74)

18 1 or 2 or 3 or 4 or 5 or 6 or 7 or 8 or 9 or 10 or 11 or 12 or 13 or 14 or 15 or 16 or 17 (24721)

19 Mobile Applications/ (984)

20 exp Computers, Handheld/ (305)

21 Smartphone/ (599)

22 Cell Phone/ (764)

23 Telemedicine/ (2617)

24 (mobile adj3 (application? or app? or device? or technolog* or computer*)).tw,kw. (5144)

25 (portable adj3 (application? or app? or device? or technolog* or computer?)).tw,kw. (813)

26 (mhealth or m-health).tw,kw. (1689)

27 ((app? or application? or device? or technolog* or platform* or computer program* or software?) adj3 (smartphone? or phone? or cellphone? or tablet? or handheld or hand-held or iphone? or ipad? or android? or touchscreen? or touch-screen?)).tw,kw. (5986)

28 ((app? or application?) adj3 computer?).tw,kw. (296)

29 or/19-28 (13981)

30 18 and 29 (185)

31 limit 30 to no language specified (95)

32 http*.so. (385189)

33 31 and 32 (84)

34 31 not 33 (11)

35 limit 30 to english language (89)

36 34 or 35 (100)

37 36 not (exp animals/ not exp humans/) (100)

***************************

Database: **Embase <1974 to 2022 February 21>**

Search Strategy:

--------------------------------------------------------------------------------

1 exp dementia/ (399577)

2 Wernicke encephalopathy/ (2918)

3 cognitive defect/ (187631)

4 dement*.tw,kw. (192647)

5 alzheimer*.tw,kw. (233751)

6 (lewy* adj2 bod*).tw,kw. (15944)

7 (chronic adj2 cerebrovascular).tw,kw. (1162)

8 ('organic brain disease' or 'organic brain syndrome').tw,kw. (1041)

9 ('normal pressure hydrocephalus' and 'shunt*').tw,kw. (2061)

10 'benign senescent forgetfulness'.tw,kw. (31)

11 (cerebr* adj2 deteriorat*).tw,kw. (353)

12 (cerebral* adj2 insufficient*).tw,kw. (109)

13 (pick* adj2 disease).tw,kw. (4209)

14 (creutzfeldt or jcd or cjd).tw,kw. (9492)

15 huntington*.tw,kw. (26578)

16 binswanger*.tw,kw. (779)

17 korsako*.tw,kw. (1833)

18 or/1-17 (613968)

19 mobile application/ (16936)

20 personal digital assistant/ (1680)

21 smartphone/ (19388)

22 mobile phone/ (19662)

23 telemedicine/ (35999)

24 (mobile adj3 (application? or app? or device? or technolog* or computer*)).tw,kw. (21669)

25 (portable adj3 (application? or app? or device? or technolog* or computer?)).tw,kw. (8014)

26 (mhealth or m-health).tw,kw. (7300)

27 ((app? or application? or device? or technolog* or platform* or computer program* or software?) adj3 (smartphone? or phone? or cellphone? or tablet? or handheld or hand-held or iphone? or ipad? or android? or touchscreen? or touch-screen?)).tw,kw. (23083)

28 ((app? or application?) adj3 computer?).tw,kw. (4966)

29 or/19-28 (113884)

30 18 and 29 (2112)

31 limit 30 to english language (2070)

32 31 not ((exp animal/ or exp invertebrate/ or nonhuman/ or animal experiment/ or animal tissue/ or animal model/ or exp plant/ or exp fungus/) not (exp human/ or human tissue/)) (2021)

33 32 not medline.cr. (1575)

34 limit 33 to dc=20191206-20220222 (632)

***************************

Database: **APA PsycInfo <1806 to February Week 2 2022>**

Search Strategy:

--------------------------------------------------------------------------------

1 organic brain syndromes/ or alzheimer's disease/ or exp dementia/ (85535)

2 wernicke's syndrome/ (284)

3 dement*.tw. (75308)

4 alzheimer*.tw. (67506)

5 (lewy* adj2 bod*).tw. (4392)

6 (chronic adj2 cerebrovascular).tw. (96)

7 ('organic brain disease' or 'organic brain syndrome').tw. (840)

8 ('normal pressure hydrocephalus' and 'shunt*').tw. (234)

9 'benign senescent forgetfulness'.tw. (28)

10 (cerebr* adj2 deteriorat*).tw. (52)

11 (cerebral* adj2 insufficient*).tw. (6)

12 (pick* adj2 disease).tw. (694)

13 (creutzfeldt or jcd or cjd).tw. (1169)

14 huntington*.tw. (5197)

15 binswanger*.tw. (505)

16 korsako*.tw. (1322)

17 or/1-16 (121555)

18 exp mobile devices/ (9662)

19 telemedicine/ (6459)

20 (mobile adj3 (application? or app? or device? or technolog* or computer*)).tw. (8023)

21 (portable adj3 (application? or app? or device? or technolog* or computer?)).tw. (737)

22 (mhealth or m-health).tw. (1613)

23 ((app? or application? or device? or technolog* or platform* or computer program* or software?) adj3 (smartphone? or phone? or cellphone? or tablet? or handheld or hand-held or iphone? or ipad? or android? or touchscreen? or touch-screen?)).tw. (5065)

24 ((app? or application?) adj3 computer?).tw. (2169)

25 or/18-24 (25286)

26 17 and 25 (382)

27 limit 26 to english language (357)

28 limit 27 to animal (11)

29 limit 28 to human (5)

30 27 not (28 not 29) (351)

31 limit 30 to up=20191206-20220222 (101)

***************************

**Scopus**
UPDATED: 2022-02-22

Total: 1,409 results

Update: 486 results

((TITLE-ABS-KEY(dement* OR alzheimer* ) OR TITLE-ABS-KEY(lewy w/2 bod*) OR TITLE-ABS-KEY(chronic w/2 cerebrovascular) OR TITLE-ABS-KEY("organic brain disease" or "organic brain syndrome") OR TITLE-ABS-KEY("normal pressure hydrocephalus" AND shunt*) OR TITLE-ABS-KEY("benign senescent forgetfulness") OR TITLE-ABS-KEY(cerebr* w/2 deteriorat*) OR TITLE-ABS-KEY(pick* w/2 disease) OR TITLE-ABS-KEY(cerebral* w/2 insufficient*) OR TITLE-ABS-KEY(creutzfeldt OR jcd OR cjd OR huntington* OR binswanger* OR korsako))) AND ((TITLE-ABS-KEY(mobile w/3 (application* or app* or device* or technolog* or computer*)) OR TITLE-ABS-KEY(portable w/3 (application* or app* or device* or technolog* or computer*)) OR TITLE-ABS-KEY(mhealth OR "m-health") OR TITLE-ABS-KEY((app* or application* or device* or technolog* or platform* or "computer program*" or software*) w/3 (smartphone* or phone* or cellphone* or tablet* or handheld or "hand-held" or iphone* or ipad* or android* or touchscreen* or "touch-screen*")) OR TITLE-ABS-KEY( (app* or application*) w/3 computer*))) AND **ORIG-LOAD-DATE > 20191205** AND ( LIMIT-TO ( LANGUAGE,"English" ) )

**Compendex**

UPDATED: 2022-02-22
Total: 1,314 results

Update: 363 kept

(((( ((((((software* NEAR/3 $smartphone) WN KY) OR ((software* NEAR/3 $phone) WN KY)) OR ((software* NEAR/3 cellphone*) WN KY))) OR ((((($computer program* NEAR/3 $smartphone) WN KY) OR (($computer program* NEAR/3 $phone) WN KY)) OR (($computer program* NEAR/3 cellphone*) WN KY))) OR (((((platform* NEAR/3 $smartphone) WN KY) OR ((platform* NEAR/3 $phone) WN KY)) OR ((platform* NEAR/3 cellphone*) WN KY))) OR (((((technolog* NEAR/3 $smartphone) WN KY) OR ((technolog* NEAR/3 $phone) WN KY)) OR ((technolog* NEAR/3 cellphone*) WN KY))) OR (((((device* NEAR/3 $smartphone) WN KY) OR ((device* NEAR/3 $phone) WN KY)) OR ((device* NEAR/3 cellphone*) WN KY)) ) OR (((((application* NEAR/3 $smartphone) WN KY) OR ((application* NEAR/3 $phone) WN KY)) OR ((application* NEAR/3 cellphone*) WN KY)) ) OR ((((($apps NEAR/3 $smartphone) WN KY) OR (($apps NEAR/3 $phone) WN KY)) OR (($apps NEAR/3 cellphone*) WN KY)) ) OR ((((($app NEAR/3 $smartphone) WN KY) OR (($app NEAR/3 $phone) WN KY)) OR (($app NEAR/3 cellphone*) WN KY))) OR ((((($app NEAR/3 $smartphone) WN KY) OR (($app NEAR/3 $phone) WN KY)) OR (($app NEAR/3 cellphone*) WN KY)) ) OR ((($mhealth OR $m-health) WN KY) ) OR ((((($computer NEAR/3 application*) WN KY) OR (($computer NEAR/3 $app) WN KY)) OR (($computer NEAR/3 $apps) WN KY)) ) OR ((((($portable NEAR/3 application*) WN KY) OR (($portable NEAR/3 $app) WN KY)) OR (($portable NEAR/3 $apps) WN KY)) ) OR ((((($mobile NEAR/3 application*) WN KY) OR (($mobile NEAR/3 $app) WN KY)) OR (($mobile NEAR/3 $apps) WN KY)) ))) AND ( ((((dement* OR alzheimer* OR $creutzfeldt OR $jcd OR $cjd OR huntington* OR binswanger* OR $korsako) WN KY) OR (($lewy NEAR/2 bod*) WN KY)) OR (($chronic NEAR/2 $cerebrovascular) WN KY))))) AND ({english} WN LA))

**Inspec**

UPDATED: 2022-02-22
Total: 834 results

Update: 254 imported

(((( ((((((software* NEAR/3 $smartphone) WN KY) OR ((software* NEAR/3 $phone) WN KY)) OR ((software* NEAR/3 cellphone*) WN KY)) AND (1884-2018 WN YR)) OR ((((($computer program* NEAR/3 $smartphone) WN KY) OR (($computer program* NEAR/3 $phone) WN KY)) OR (($computer program* NEAR/3 cellphone*) WN KY)) AND (1884-2018 WN YR)) OR (((((platform* NEAR/3 $smartphone) WN KY) OR ((platform* NEAR/3 $phone) WN KY)) OR ((platform* NEAR/3 cellphone*) WN KY)) AND (1884-2018 WN YR)) OR (((((technolog* NEAR/3 $smartphone) WN KY) OR ((technolog* NEAR/3 $phone) WN KY)) OR ((technolog* NEAR/3 cellphone*) WN KY)) AND (1884-2018 WN YR)) OR (((((device* NEAR/3 $smartphone) WN KY) OR ((device* NEAR/3 $phone) WN KY)) OR ((device* NEAR/3 cellphone*) WN KY)) AND (1884-2018 WN YR)) OR (((((application* NEAR/3 $smartphone) WN KY) OR ((application* NEAR/3 $phone) WN KY)) OR ((application* NEAR/3 cellphone*) WN KY)) AND (1884-2018 WN YR)) OR ((((($apps NEAR/3 $smartphone) WN KY) OR (($apps NEAR/3 $phone) WN KY)) OR (($apps NEAR/3 cellphone*) WN KY)) AND (1884-2018 WN YR)) OR ((((($app NEAR/3 $smartphone) WN KY) OR (($app NEAR/3 $phone) WN KY)) OR (($app NEAR/3 cellphone*) WN KY)) AND (1884-2018 WN YR)) OR ((((($app NEAR/3 $smartphone) WN KY) OR (($app NEAR/3 $phone) WN KY)) OR (($app NEAR/3 cellphone*) WN KY)) AND (1884-2018 WN YR)) OR ((($mhealth OR $m-health) WN KY) AND (1884-2018 WN YR)) OR ((((($computer NEAR/3 application*) WN KY) OR (($computer NEAR/3 $app) WN KY)) OR (($computer NEAR/3 $apps) WN KY)) AND (1884-2018 WN YR)) OR ((((($portable NEAR/3 application*) WN KY) OR (($portable NEAR/3 $app) WN KY)) OR (($portable NEAR/3 $apps) WN KY)) AND (1884-2018 WN YR)) OR ((((($mobile NEAR/3 application*) WN KY) OR (($mobile NEAR/3 $app) WN KY)) OR (($mobile NEAR/3 $apps) WN KY)) AND (1884-2018 WN YR)))) AND ( ((((dement* OR alzheimer* OR $creutzfeldt OR $jcd OR $cjd OR huntington* OR binswanger* OR $korsako) WN KY) OR (($lewy NEAR/2 bod*) WN KY)) OR (($chronic NEAR/2 $cerebrovascular) WN KY)) AND (1884-2018 WN YR)))) AND ({english} WN LA))

**--
Proquest Dissertations and Theses Global**

UPDATED: 2022-02-22
Total: 59 results

Update: 21 kept

(noft(dement* OR alzheimer* OR creutzfeldt OR jcd OR cjd OR huntington* OR binswanger* OR korsako) OR noft(lewy NEAR/2 bod*) OR noft(chronic NEAR/2 cerebrovascular) OR noft("organic brain disease" OR "organic brain syndrome") OR (normal pressure hydrocephalus AND shunt*) OR "'benign senescent forgetfulness" OR (cerebr* NEAR/2 deteriorat*) OR (cerebral* NEAR/2 insufficient*) AND (pick* NEAR/2 disease)) AND (noft(mobile NEAR/3 (application* OR app OR apps OR device* OR technolog* OR computer*)) OR noft(portable NEAR/3 (application* OR app OR apps OR device* OR technolog* OR computer*)) OR noft(mhealth OR m-health) OR noft(((app OR apps OR application* OR device* OR technolog* OR platform* OR "computer program*" OR software*) NEAR/3 (smartphone* OR phone* OR cellphone* OR tablet* OR handheld OR hand-held OR iphone* OR ipad* OR android* OR touchscreen* OR touch-screen*))) OR noft((app OR apps OR application*) NEAR/3 computer*))

*Limited to English using limits post-search

**ACM Digital Library**

UPDATED: 2019.12.09
Total: 665 results
Update: 195 kept

(Title:(app apps application* mobile portable mhealth "m-health" technolog* computer* smartphone* or cellphone* phone* ipad* iphone* android* device* software* platform*) OR Abstract:(app apps application* mobile portable mhealth "m-health" technolog* computer* smartphone* or cellphone* phone* ipad* iphone* android* device* software* platform*)) AND (Title:(dement* alzheimer* creutzfeldt jcd cjd huntington* binswanger* korsako "lewy body" "lewy bodies" "chronic cerebrovascular" "organic brain disease" "organic brain syndrome*" "benign senescent forgetfulness" "pick disease") OR Abstract:((dement* alzheimer* creutzfeldt jcd cjd huntington* binswanger* korsako "lewy body" "lewy bodies" "chronic cerebrovascular" "organic brain disease" "organic brain syndrome*" "benign senescent forgetfulness" "pick disease"))
