# Supplementary material for: Researched Apps Used in Dementia Care for People Living With Dementia and Their Informal Caregivers: Systematic Review on App Features, Security, and Usability
Source: J Med Internet Res. 2023 Oct 12;25:e46188. doi: 10.2196/46188 (PMC10603562; doi:10.2196/46188)
Supplement: Multimedia Appendix 3 [file jmir_v25i1e46188_app3.docx]

| Table S1. A description of study and app characteristics | | | | | | | | | |
| --- | --- | --- | --- | --- | --- | --- | --- | --- | --- |
| **Author name** | **Publication year** | **Country** | **Funding information** | **Publication channel** | **App name** | **App commercially available** | **Device type** | **OS** | **App developed for** |
| Aljehani, S., et al. | 2018 | Saudi Arabia | DNM | Conference paper | iCare | No | Smartphone – iPhone | iOS | ICG |
| Asghar, I., et al. | 2020 | UK, China | Yes | Journal | Assistive Brotherhood Community (ABC) application | No | Tablet, smartphone | Android | PLwD |
| Boyd, A., et al. | 2017 | UK | Yes | Journal | Brain fit plan (BFP) | No | Tablet | Android | PLwD |
| Boyd, K., et al. | 2021 | UK | Yes | Journal | InspireD | Yes | Tablet | iOS and Android | PLwD |
| Brown, E., et al. | 2016 | US | Yes | Journal | CareHeroes | Yes | Smartphone | Android and web-based | ICG |
| Brown, J., et al. | 2020 | US | DNM | Conference paper | DNM | No | Did not use any | DNM | ICG |
| Chaudhry, B. & Smith, J. | 2021 | US | DNM | Conference paper | RefineMind | No | DNM | DNM | PLwD and ICG |
| Critten, V. & Kucirkova, N. | 2017 | UK | No | Journal | Our story | Yes | Tablet - iPad | iOS and Android | PLwD |
| Ekstrom, A., et al. | 2017 | Sweden | Yes | Journal | GoTalk NOW | Yes | Tablet | iOS, Android, web-based, Windows and Mac | PLwD |
| EI Haj, M., et al. | 2017 | France | Yes | Journal | Google calendar | Yes | Smartphone | iOS and Android | PLwD |
| Evans, N., et al. | 2021 | UK | Yes | Journal | DNM | No | Tablet | DNM | PLwD |
| Favilla, S. & Pedell, S. | 2013 | Australia | DNM | Conference paper | Touch OSC | Yes | Tablet - iPad | iOS | PLwD |
| Gibson, A., et al. | 2016 | Ireland | DNM | Conference paper | InspireD | Yes | Tablet - iPad | iOS and Android | PLwD |
| Groenewoud, H., et al. | 2017 | Netherland, UK, Canada | DNM | Journal | 10 commercially game apps and 3 new game apps (Shopping, Pets and Soccer) | 10 existing game apps – yes;  Newly developed apps – no; | Tablet - iPad | iOS and Android | PLwD |
| Hackett, K., et al. | 2022 | US | Yes | Journal | SmartPrompt | No | Smartphone | iOS and Android | PLwD |
| Hashim-de Vries, A., et al. | 2018 | Malaysia | DNM | Conference paper | my-MOBAL - P20 | No | Tablet | Android | PLwD |
| Hassan, N., et al. | 2021 | Malaysia | DNM | Journal | Diary | No | DNM | DNM | PLwD |
| Hettinga, M., et al. | 2009 | Netherland, US | DNM | Journal | TomTom | Yes | Smartphone | iOS and Android | PLwD |
| Hughes, J., et al. | 2020 | US | Yes | Journal | CAST 2.0 | No | DNM | Android | ICG |
| Imbeault, H., et al. | 2014 | Canada | DNM | Journal | AP@LZ | No | Smartphone | Windows | PLwD |
| Kelleher, J., et al. | 2021 | US | Yes | Journal | MapHabit | Yes | Tablet -iPad | iOS and Android | PLwD |
| Lai, R., et al. | 2020 | Australia, Germany | Yes | Journal | DNM | No | DNM | DNM | PLwD |
| Manera, V., et al. | 2015 | France | Yes | Journal | Kitchen and cooking -serious game app | No | Tablet - iPad | iOS | PLwD |
| McCarron, H., et al. | 2019 | US | Yes | Journal | Social support aid (SSA); | No | Smartphone | DNM | PLwD |
| McCauley, C., et al. | 2019 | UK | Yes | Journal | InspireD | Yes | Tablet -iPad | iOS and Android | PLwD |
| Morrissey, K., et al. | 2017 | UK | Yes | Conference paper | Care and Connect | No | Tablet | iOS and Android | PLwD and ICG |
| Oksnebjerg, L., Woods, B, Vilsen, C.R., et al. | 2020 | Denmark, UK, | DNM | Journal | ReACT | No | Tablet -iPad | iOS | PLwD |
| Oksnebjerg, L., Woods, B, Ruth, K., et al. | 2020 | Denmark, UK, | DNM | Journal | ReACT | No | Tablet -iPad | iOS | PLwD |
| Pirani, E., et al. | 2016 | India | DNM | Conference paper | DNM | No | Smartphone | Android | PLwD |
| Quintana, M., et al. | 2020 | Spain, Sweden, UK | Yes | Journal | SMART4MD | No | Tablet | Android | PLwD |
| Rai, K. H., Prasetya, V.G.H., et al. | 2021 | UK, Indonesia | Yes | Journal | iCST/Thinkability | Yes | Tablet | iOS and Android | PLwD and ICG |
| Rai, K. H., Griffiths, R., et al. | 2021 | UK | Yes | Journal | iCST/Thinkability | Yes | Tablet | iOS and Android | PLwD and ICG |
| Rettinger, L., et al. | 2020 | Austria | Yes | Conference paper | DEA | No | Smartphone - Android, tablet | Android | ICG |
| Reyes, A., et al. | 2016 | Colombia | Yes | Conference paper | DNM | No | Smartphone, tablet | DNM | PLwD and ICG |
| Ruggiano, N., et al. | 2019 | US | DNM | Journal | CareHeroes | Yes | Smartphone - Android | Android | ICG |
| Ryan, A., et al. | 2018 | Northern Ireland | Yes | Journal | InspireD | Yes | Tablet - iPad | iOS and Android | PLwD |
| Savita, K.S., et al. | 2019 | Malaysia | Yes | Conference paper | MyDem | No | DNM | Android | PLwD |
| Schultz, T., et al. | 2021 | Germany | Yes | Journal | I-Care | No | Tablet | Android | PLwD and ICG |
| Siddiq, K., et al. | 2018 | Pakistan | DNM | Journal | CareD | No | Smartphone | Android | PLwD and ICG |
| Tyack, C., et al. | 2017 | UK | Yes | Journal | Art-viewing app | No | Tablet | Android | PLwD |
| Welsh, D., et al. | 2018 | UK | DNM | Conference paper | Ticket to Talk | No | Tablet, smartphone | iOS and Android | ICG |
| Wu, P.F., et al. | 2020 | China (Taiwan) | Yes | Journal | Recall | No | DNM | DNM | PLwD |
| Yamagata, C., et al. | 2013 | US | Yes | Conference paper | Jungle app | No | Tablet - iPad | DNM | PLwD |
| Yu, F., et al. | 2019 | US | Yes | Journal | Memory Matters | Yes | Tablet - iPad | iOS | PLwD |

DNM: Did not mention

ICG: Informal caregiver

PLwD: People living with dementia
