# Supplementary material for: Researched Apps Used in Dementia Care for People Living With Dementia and Their Informal Caregivers: Systematic Review on App Features, Security, and Usability
Source: J Med Internet Res. 2023 Oct 12;25:e46188. doi: 10.2196/46188 (PMC10603562; doi:10.2196/46188)
Supplement: Multimedia Appendix 4 [file jmir_v25i1e46188_app4.docx]

**Supplement B – Quality assessment appraisal tools used corresponding to each study**

**CEBM case study appraisal questions**

1. Did the study address a clearly focused question / issue?

2. Is the research method (study design) appropriate for answering the research question?

3. Are both the setting and the subjects representative with regard to the population to which the findings will be referred?

4. Is the researcher’s perspective clearly described and taken into account?

5. Are the methods for collecting data clearly described?

6. Are the methods for analyzing the data likely to be valid and reliable? Are quality control measures used?

7. Was the analysis repeated by more than one researcher to ensure reliability?

8. Are the results credible, and if so, are they relevant for practice?

9. Are the conclusions drawn justified by the results?

10. Are the findings of the study transferable to other settings?

|  | **Q1** | **Q2** | **Q3** | **Q4** | **Q5** | **Q6** | **Q7** | **Q8** | **Q9** | **Q10** |
| --- | --- | --- | --- | --- | --- | --- | --- | --- | --- | --- |
| Critten, V. & Kucirkova, N., 2017 | Yes | Yes | Yes | Yes | Yes | Yes | Yes | Yes | Yes | Yes |
| Haj, EI. M., et al., 2017 | Yes | Yes | Yes | Yes | Yes | No | No | Yes | Yes | Yes |
| Ekstrom, A., et al., 2017 | Yes | Yes | Yes | Yes | Yes | Yes | No | Yes | Yes | Yes |
| Imbeault, H., et al., 2014 | Yes | Yes | Yes | Yes | Yes | No | No | Yes | Yes | Yes |
| Hashim-de Vries, A., et al., 2018 | No | Yes | Yes | No | No | No | Can’t tell | Yes | Yes | Yes |

**MMAT mixed methods study appraisal questions**

1. Are there clear research questions?

2. Do the collected data allow to address the research questions?

3. Is there an adequate rationale for using a mixed methods design to address the research question?

4. Are the different components of the study effectively integrated to answer the research question?

5. Are the outputs of the integration of qualitative and quantitative components adequately interpreted?

6. Are divergences and inconsistencies between quantitative and qualitative results adequately addressed?

7. Do the different components of the study adhere to the quality criteria of each tradition of the methods involved?

|  | **Q1** | **Q2** | **Q3** | **Q4** | **Q5** | **Q6** | **Q7** |
| --- | --- | --- | --- | --- | --- | --- | --- |
| Brown, E., et al., 2016 | Yes | Yes | No | Yes | Yes | Can’t tell | Yes |
| Gibson, A., et al., 2016 | Yes | Yes | No | Yes | Yes | Yes | Yes |
| Groenewoud, H., et al., 2017 | Yes | Yes | No | Yes | No | No | Yes |
| Reyes, A., et al., 2016 | No | No | No | No | No | No | Can’t tell |
| Tyack, C., et al., 2017 | Yes | Yes | No | Yes | Yes | Yes | Yes |
| Oksnebjerg, L., et al., 2019 | Yes | Yes | No | No | No | No | Yes |
| McCauley, C., et al., 2019 | Yes | Yes | No | Yes | Yes | Yes | Yes |
| Asghar, I., et al., 2020 | Yes | Yes | No | Yes | No | No | Yes |
| Boyd, K., et al., 2021 | Yes | Yes | No | Yes | No | No | Yes |
| Brown, J., et al., 2020 | Yes | Yes | No | Yes | Yes | No | Yes |
| Chaudhry, B. & Smith, J., 2021 | Yes | Can’t tell | No | Yes | Yes | No | Yes |
| Hackett, K., et al., 2022 | Yes | Can’t tell | No | Yes | Yes | No | Yes |
| Oksnebjerg, L., et al., 2020 | Yes | Yes | No | Yes | No | No | Yes |
| Quintana, M., et al., 2020 | Yes | Yes | No | Yes | No | No | Yes |
| Rai, K. H., Prasetya, V., et al., 2021 | Yes | Yes | Yes | Yes | No | No | Yes |
| Rai, K. H., Griffiths, R., et al., 2021 | Yes | Yes | No | Yes | No | No | Yes |
| Rettinger, L., et al., 2020 | Yes | Yes | Yes | Yes | No | No | Yes |
| Schultz, T., et al., 2021 | Yes | Yes | No | Yes | No | No | Yes |

**MMAT qualitative study appraisal questions**

1. Are there clear research questions?

2. Do the collected data allow to address the research questions?

3. Is the qualitative approach appropriate to answer the research question?

4. Are the qualitative data collection methods adequate to address the research question?

5. Are the findings adequately derived from the data?

6. Is the interpretation of results sufficiently substantiated by data?

7. Is there coherence between qualitative data sources, collection, analysis and interpretation?

|  | **Q1** | **Q2** | **Q3** | **Q4** | **Q5** | **Q6** | **Q7** |
| --- | --- | --- | --- | --- | --- | --- | --- |
| Boyd, A., et al., 2017 | Yes | Yes | Yes | Yes | Yes | Yes | Yes |
| Evans, N., et al., 2021 | Yes | Yes | Yes | Yes | Yes | Yes | Yes |
| Favilla, S. & Pedell, S., 2013 | No | Can’t tell | Yes | Yes | Yes | Yes | Yes |
| Hughes, J., et al., 2021 | Yes | Yes | Yes | Yes | Yes | Yes | Yes |
| Morrissey, K., et al., 2017 | No | No | Yes | Yes | Yes | Yes | Yes |
| Ruggiano, N., et al., 2019 | Yes | Yes | Yes | Yes | Yes | Yes | Yes |
| Ryan, A., et al., 2018 | Yes | Can’t tell | Yes | Yes | Yes | Yes | Yes |
| Welsh, D., et al., 2018 | Yes | Yes | Yes | Yes | Yes | Yes | Yes |
| Yamagata, C., et al., 2013 | No | No | Can’t tell | Can’t tell | Can’t tell | Can’t tell | Can’t tell |

**MMAT quantitative study appraisal questions**

1. Are there clear research questions?

2. Do the collected data allow to address the research questions?

3. Is the sampling strategy relevant to address the research question?

4. Is the sample representative of the target population?

5. Are the measurements appropriate?

6. Is the risk of nonresponse bias low?

7. Is the statistical analysis appropriate to answer the research question?

|  | **Q1** | **Q2** | **Q3** | **Q4** | **Q5** | **Q6** | **Q7** |
| --- | --- | --- | --- | --- | --- | --- | --- |
| Aljehani, S., et al., 2018 | Yes | Can’t tell | Can’t tell | Can’t tell | Can’t tell | Can’t tell | Can’t tell |
| Pirani, E., et al., 2016 | No | No | No | Can’t tell | Can’t tell | Can’t tell | Yes |
| Savita, K.S., et al., 2019 | Yes | Yes | No | Can’t tell | Yes | Can’t tell | Yes |
| Siddiq, K., et al., 2018 | Yes | Yes | No | Can’t tell | Can’t tell | Can’t tell | Can’t tell |

**MMAT random control trial study appraisal questions**

1. Are there clear research questions?

2. Do the collected data allow to address the research questions?

3. Is randomization appropriately performed?

4. Are the groups comparable at baseline?

5. Are there complete outcome data?

6. Are outcome assessors blinded to the intervention provided?

7. Did the participants adhere to the assigned intervention?

|  | **Q1** | **Q2** | **Q3** | **Q4** | **Q5** | **Q6** | **Q7** |
| --- | --- | --- | --- | --- | --- | --- | --- |
| Hassan, N., et al., 2021 | Yes | Can’t tell | Can’t tell | Yes | Yes | Can’t tell | Yes |
| Hettinga, M., et al., 2009 | No | Can’t tell | Can’t tell | Can’t tell | Yes | Can’t tell | Yes |
| McCarron, H., et al., 2019 | Yes | Can’t tell | Yes | Yes | Yes | No | Yes |
| Yu, F., et al., 2019 | Yes | Can’t tell | Yes | Yes | Yes | Yes | Yes |

**MMAT non-random control trial study appraisal questions**

1. Are there clear research questions?

2. Do the collected data allow to address the research questions?

3. Are the participants representative of the target population?

4. Are measurements appropriate regarding both the outcome and intervention (or exposure)?

5. Are there complete outcome data?

6. Are the confounders accounted for in the design and analysis?

7. During the study period, is the intervention administered (or exposure occurred) as intended?

|  | **Q1** | **Q2** | **Q3** | **Q4** | **Q5** | **Q6** | **Q7** |
| --- | --- | --- | --- | --- | --- | --- | --- |
| Kelleher, J., et al., 2021 | Yes | Yes | Yes | Yes | Yes | Can’t tell | Yes |
| Lai, R., et al., 2020 | Yes | Yes | Yes | Yes | Yes | Can’t tell | Yes |
| Manera, V., et al., 2015 | Yes | Yes | Yes | Yes | Yes | Can’t tell | Yes |
| Wu, P.F., et al., 2020 | Yes | Yes | Yes | Yes | Yes | Can’t tell | Yes |
