# Supplementary material for: Researched Apps Used in Dementia Care for People Living With Dementia and Their Informal Caregivers: Systematic Review on App Features, Security, and Usability
Source: J Med Internet Res. 2023 Oct 12;25:e46188. doi: 10.2196/46188 (PMC10603562; doi:10.2196/46188)
Supplement: Multimedia Appendix 5 [file jmir_v25i1e46188_app5.docx]

Table S2. A description of participant characteristics

| **Author name** | **Participant type** | **Sample size for PLwD** | **Sample size for ICG** | **Age (mean) PLwD** | **Age (range) PLwD** | **Age (mean) ICG** | **Age (range) ICG** | **Reported previous computer experience** |
| --- | --- | --- | --- | --- | --- | --- | --- | --- |
| Aljehani, S., et al. | ICG | Na | 36 | Na | Na | DNM | DNM | DNM |
| Asghar, I., et al. | Mild PLwD and ICG | 8 | 40 | 70.3 | 66-74 | DNM | DNM | Yes |
| Boyd, A., et al. | Early stage of PLwD | 6 | Na | 82 | DNM | Na | Na | Yes |
| Boyd, K., et al. | Mild and moderate PLwD and ICG | 9 | 5 | 69.3 | 60-81 | DNM | DNM | Yes |
| Brown, E., et al. | ICG | Na | 11 | Na | Na | 56.5 | DNM | Yes |
| Brown, J., et al. | ICG | Na | 20 | Na | Na | DNM | DNM | Yes |
| Chaudhry, B. & Smith, J. | ICG | Na | 5 | Na | Na | DNM | DNM | DNM |
| Critten, V. & Kucirkova, N. | Mild to moderate PLwD | 3 | Na | 83.3 | 72-94 | Na | Na | DNM |
| Ekstrom, A., et al. | AD and her husband | 1 | 1 | 52 | 52 | DNM | DNM | Yes |
| EI Haj, M., et al. | Mild AD and her husband | 1 | 1 | 66 | 66 | DNM | DNM | Yes |
| Evans, N., et al. | Mild to moderate PLwD and ICG | 26 | 26 | 80 | 67-94 | DNM | DNM | DNM |
| Favilla, S. & Pedell, S. | PLwD | 12 | Na | DNM | DNM | Na | Na | DNM |
| Gibson, A., et al. | PLwD and ICG | 7 | 7 | DNM | DNM | DNM | DNM | Yes |
| Groenewoud, H., et al. | Severe, and mild to moderate PLwD | 54 | Na | 83.5 | 59-95 | Na | Na | Yes |
| Hackett, K., et al. | PLwD and ICG | 10 | 10 | 80.3 | 67-93 | 68 | 57-84 | Yes |
| Hashim-de Vries, A., et al. | Mild AD | 1 | Na | 74 | 74 | Na | Na | Yes |
| Hassan, N., et al. | Early stage of PLwD | 4 | Na | 65.25 | 64-67 | Na | Na | DNM |
| Hettinga, M., et al. | Mild PLwD | 4 | Na | DNM | DNM | Na | Na | DNM |
| Hughes, J., et al. | ICG | Na | 10 | Na | Na | 59 | DNM | DNM |
| Imbeault, H., et al. | Early and mild PwAD and ICG | 2 | 2 | 75.5 | 71-80 | DNM | DNM | Yes |
| Kelleher, J., et al. | People with cognitive impairment (CI) | 22 (note, it is for people with CI) | Na | DNM | DNM | Na | Na | DNM |
| Lai, R., et al. | Mild PLwD and ICG | 18 | 24 | 74.5 | 57-84 | 62.4 | 31-83 | Yes |
| Manera, V., et al. | AD or related disorders | 12 | Na | 80.3 | 70-90 | Na | Na | DNM |
| McCarron, H., et al. | PLwD and ICG | 29 | 35 | DNM | DNM | 67.8 | DNM | DNM |
| McCauley, C., et al. | Mild to moderate PLwD and ICG | 28 | 28 | 79 | 61-94 | 67 | 31-91 | Yes |
| Morrissey, K., et al. | PLwD and ICG | 2 | 28 | DNM | DNM | DNM | DNM | DNM |
| Oksnebjerg, L., Woods, B., Wilsen, C.R., et al. | Mild AD and ICG | 19 | 19 | 67.5 | 52-79 | 67.3 | 51-83 | Yes |
| Oksnebjerg, L., Woods, B., Ruth, K., et al. | Early stage of PLwD and ICG | 112 | 98 | 68 | 39-86 | DNM | DNM | Yes |
| Pirani, E., et al. | DNM | Na | Na | Na | Na | Na | Na | DNM |
| Quintana, M., et al. | Mild PLwD and ICG | 19 | 19 | 78.5 | DNM | 66 | DNM | Yes |
| Rai, K. H., Prasetya, V.G.H., et al. | PLwD and ICG | 6 | 12 | 74.5 | 60-83 | 53.3 | 35-72 | Yes |
| Rai, K. H., Griffiths, R., et al. | Mild to moderate PLwD and ICG | 29 | 31 | 73.9 | 67-86 | 68.5 | 49-82 | Yes |
| Rettinger, L., et al. | ICG | Na | 15 | Na | Na | 49 | 17-71 | Yes |
| Reyes, A., et al. | ICG of early and moderate AD | Na | 5 | Na | Na | 41 | 29-50 | Yes |
| Ruggiano, N., et al. | ICG | Na | 36 | Na | Na | 65.7 | 42-89 | DNM |
| Ryan, A., et al. | Mild to moderate PLwD and ICG | 15 | 17 | 78.1 | 61-94 | 69.1 | 31-91 | DNM |
| Savita, K.S., et al. | Early and mild PLwD | 4 | Na | DNM | 41-65 | Na | Na | DNM |
| Schultz, T., et al. | PLwD | 29 | Na | 82.2 | DNM | Na | Na | DNM |
| Siddiq, K., et al. | Mild and moderate PwAD and ICG | 70 | DNM | DNM | DNM | DNM | DNM | DNM |
| Tyack, C., et al. | PLwD and ICG | 12 | 12 | 75 | 64-90 | 66 | 48-77 | Yes |
| Welsh, D., et al. | PLwD and ICG | 1 | 15 | DNM | DNM | DNM | 65-80 | DNM |
| Wu, P., et al. | Mild PLwD | 16 | Na | 79.5 | 65-94 | Na | Na | Yes |
| Yamagata, C., et al. | PLwD | DNM | Na | DNM | DNM | Na | Na | DNM |
| Yu, F., et al. | PLwD and ICG | 80 | DNM | 82.1 | 62-98 | DNM | DNM | DNM |

Na: not applicable

DNM: Did not mention

ICG: Informal caregiver

PLwD: People living with dementia

PwAD: People with Alzheimer’s disease
