# Supplementary figures and images for: Researched Apps Used in Dementia Care for People Living With Dementia and Their Informal Caregivers: Systematic Review on App Features, Security, and Usability
Source: J Med Internet Res. 2023 Oct 12;25:e46188. doi: 10.2196/46188 (PMC10603562; doi:10.2196/46188)

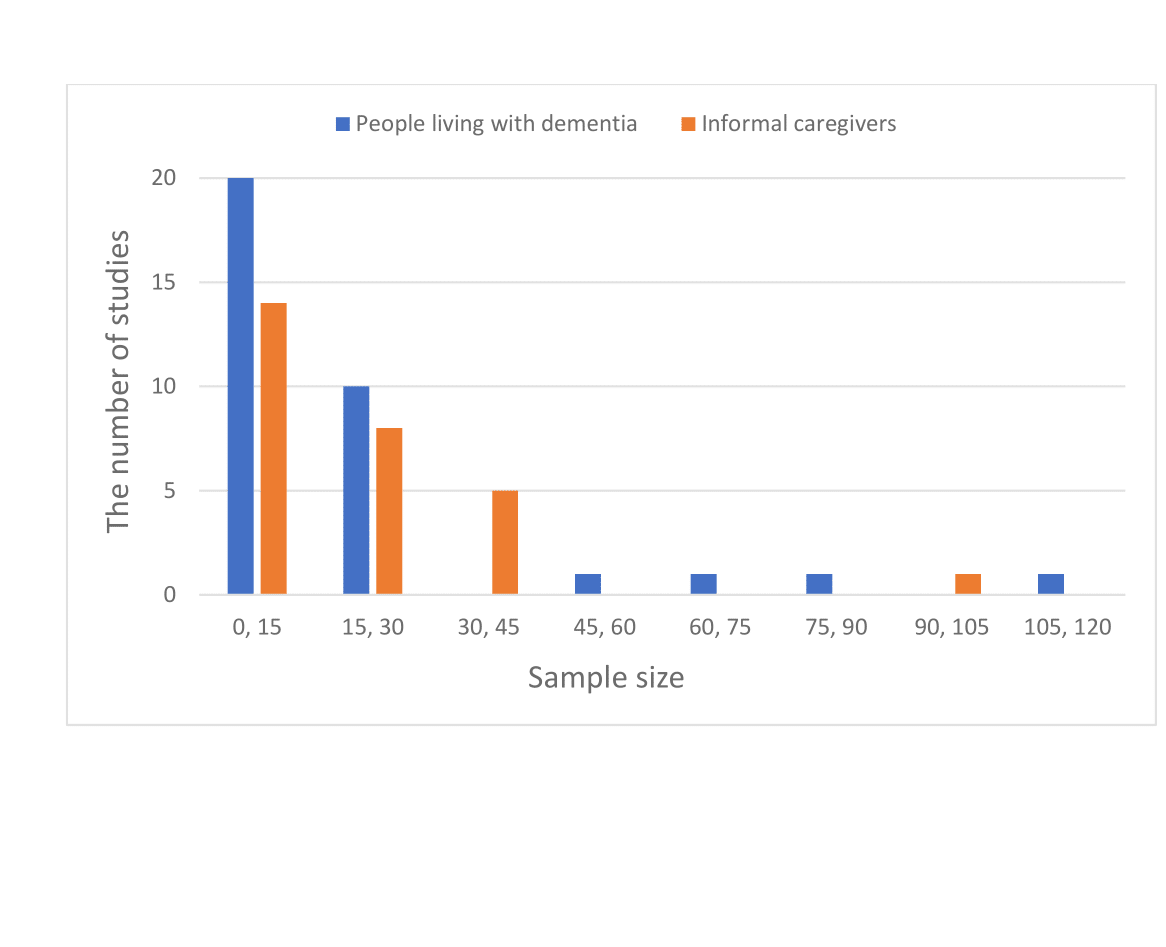

Supplement: Multimedia Appendix 6 [file jmir_v25i1e46188_app6.png]
