# Supplementary material for: Researched Apps Used in Dementia Care for People Living With Dementia and Their Informal Caregivers: Systematic Review on App Features, Security, and Usability
Source: J Med Internet Res. 2023 Oct 12;25:e46188. doi: 10.2196/46188 (PMC10603562; doi:10.2196/46188)
Supplement: Multimedia Appendix 7 [file jmir_v25i1e46188_app7.docx]

Table S3. A summary of app features for PLwD across the studies, ranked in descending order of occurrence.

| **App features** |
| --- |
| Cognitive stimulation – Includes: reminiscence, brain games and quizzes (n=17) |
| Reminder or step-by-step prompt to complete ADLs (n=13) |
| Social support – Includes: 1) Connection; 2) Recognition; 3) Calling help; 4) Needs support; 5) Communication aid and 6) Location-based review (n=12) |
| Scheduling for PLwD (n=7) |
| Navigation (n=5) |
| Orientation of time – Provides today’s day, date or time. (n=5) |
| Leisure activities – Includes: games, diary writing, music creation and arts viewing. (n=5) |
| PLwD’s progress report – Includes such as PLwD’s performance on the memory games. (n=4) |
| Resources for PLwD - Includes recommended exercise and a list of rehab centers. (n=3) |
| Personalization – Apps could be customized based on personal preference, such as font size, background image, and app appearance. (n=3) |
| Health record - Regards of PLwD’s medication and health (i.e., symptoms) information. (n=2) |
| Alarm – Generated automatically to remind PLwD to complete an unfinished task or to warn PLwD to go back home if they are outside the specified home range. (n=2) |
| Reward – Motivates PLwD to use the app. (n=2) |
| Mood tracker – Tracks PLwD’s mood by recording it to the app using either typing or voice recording. (n=1) |
| News and weather updates (n=1) |
| Guide – Navigates users to use the app (n=1) |

ADL: Activity of daily living

n is the number of studies
