# Supplementary material for: Researched Apps Used in Dementia Care for People Living With Dementia and Their Informal Caregivers: Systematic Review on App Features, Security, and Usability
Source: J Med Internet Res. 2023 Oct 12;25:e46188. doi: 10.2196/46188 (PMC10603562; doi:10.2196/46188)
Supplement: Multimedia Appendix 8 [file jmir_v25i1e46188_app8.docx]

Table S4. A summary of app features for caregivers across the studies ranked in descending order of occurrence.

| **App features** | **Description** |
| --- | --- |
| Activities to engage both PLwD and their caregivers (n=4) | These apps provide suitable activities that can be completed by PLwD and their caregivers together, such as playing a game together or a mutual task like caring for flowers. |
| Caregiving support (n=4) | These apps provide information and support for caregiving activities, such as caregiving tips or frequently asked questions; OR contact information of service facilities and self-help groups for caregivers; OR connections to other caregivers for knowledge sharing about tasks and how to optimize daily care. |
| Conversation stimulation (n=3) | These apps provided various conversation topics for discussion between caregivers and the PLwD. |
| PLwD’s progress report (n=3) | These apps generated “reports” for caregivers to review, such as PLwD’s performance on the memory games to monitor the PLwD. |
| Health record (n=3) | Caregivers could use the apps to maintain or review PLwD’s medication list, intake history, intake time and dosage; OR to display PLwD’s physiological information (e.g., heart rate). |
| Caregiver support (n=3) | Four types of support: Healthy living - Educational information about caregiver wellness, healthy diet, and recommended workouts or exercises.  Emotional support – assess caregiver emotion, stress and cognitive function.  Alert/notification features – Sends caregivers resources to help reduce burden and depression symptoms; notifies care professionals to contact caregivers for any dementia-related issues/questions they may have.  Reminder – Reminds appointments or medication uptake. |
| PLwD location tracking (n=2) | Tracks PLwD’s current location. |
| Scheduling (n=2) | Adds appointments/to-do lists for PLwD. |
| Location-based review (n=1) | Provides a review and rating of a location. |
| Guide (n=1) | Navigates users to use the app |
| Personalization (n=1) | Users could customize the app, such as font size, background image, and app appearance. |
| Rewards (n=1) | Rewards were provided to motivate the user to use the app by providing information related to caregivers and PLwD. |
| Games (n=1) | A leisure activity for caregivers. |
| Alert (n=1) | Notifies caregivers when PLwD misses their scheduled appointments. |

n is the number of studies
