# Supplementary material for: Researched Apps Used in Dementia Care for People Living With Dementia and Their Informal Caregivers: Systematic Review on App Features, Security, and Usability
Source: J Med Internet Res. 2023 Oct 12;25:e46188. doi: 10.2196/46188 (PMC10603562; doi:10.2196/46188)
Supplement: Multimedia Appendix 9 [file jmir_v25i1e46188_app9.docx]

| Table S5. Privacy policy themes identified in the privacy policies from eight commercially available apps | |
| --- | --- |
| **Privacy policy themes** | **Description** |
| Contact information of developers | Provided for users with questions, comments, or concerns regarding the privacy policy. |
| Data entered by end-users | Information includes user name, password, photos, video clips, e-mail address and/or phone number. |
| Data collected through apps | Identified specific information that is automatically collected through the use of an app, such as internet protocol (IP) addresses, geolocation, viewed browsers, mobile device types, viewed videos, and search history. |
| Privacy policy changes | Informs users about any changes to a privacy policy. For example, a notification could be sent out to the users to notify the change; The changes could be announced on the privacy policy page with a revised date and version. The old privacy policy could also be saved in an archived folder for review. |
| End-users share their own data | App users share the data with a third party. The data could include users' videos and/or photos, and users’ comments. |
| App owner shares end-users data | App owners will share users’ data with a third party. |
| Procedures for misuse of data | Indicates measures to be taken when data are misused by the app owners, such as deleting any misused information. |
| User’s data control | States how users can manage their data and privacy, such as whether they can delete or update their personal information. |
| User’s privacy right | States user’s rights to control their privacy. For example, a user should have the right to limit how their personal information is being used; and the right to complain to local data protection authorities about privacy concerns. |
| Data retention | Outlines how long the app owner would store the data, if any data continue to be used even after data deletion or account deactivation, and its reasons (i.e., legal purposes) for the use. |
| Data ownership | States the app and data owners, often appearing at the privacy policy's beginning. |
| Security features | Emphasizes any security measures (e.g., encryption, security checkup, SSL, and secure data storage technologies) applied to data, especially for identifiable data. |
| Data use | Outlines how collected data will be used: user registration, research and publication purposes, legal requirements, operation management, analytics, service enhancement, new service development (i.e., users’ habit of organizing the photos), personalized advertisement creation, and product quality improvement. |
| Data storage | Indicates where data are stored, such as on a secure cloud server, backup archives, and/or locally on the device. |
| Data transfer | States whether data will be transferred to another country for storage. For example, servers may be located in different countries, resulting in data being transferred and stored in a foreign country. |
| Age restriction | States any age limits of the users for use of the app. |
| Jurisdictional Data Protection Laws, such as General Data Protection Regulation (GDPR) | Indicates whether a privacy policy is created according to a data protection law, such as GDPR. |
