# Supplementary material for: Researched Apps Used in Dementia Care for People Living With Dementia and Their Informal Caregivers: Systematic Review on App Features, Security, and Usability
Source: J Med Internet Res. 2023 Oct 12;25:e46188. doi: 10.2196/46188 (PMC10603562; doi:10.2196/46188)
Supplement: Multimedia Appendix 10 [file jmir_v25i1e46188_app10.docx]

| Table S6. Tally of themes in each app privacy policy | | | | | | | | |
| --- | --- | --- | --- | --- | --- | --- | --- | --- |
| **_Apps_**  **_Themes_** | **GoTalk NOW** | **Care Heroes** | **Google Calendar** | **InspireD** | **Memory Matters** | **Our Story** | **TomTom** | **MapHabit** |
| Contact information of developers | 1 | 1 | 1 | 1 | 1 | 1 | 1 | 1 |
| Data entered by end-users | 1 | 1 | 1 | 1 | 1 | 1 | 1 | 1 |
| Data collected through apps | 1 |  | 1 |  | 1 |  | 1 | 1 |
| Privacy policy changes | 1 | 1 | 1 | 1 | 1 |  |  | 1 |
| End-user shares their own data |  | 1 | 1 |  | 1 |  | 1 | 1 |
| App owner shares end-users’ data |  | 1 | 1 | 1 | 1 |  | 1 | 1 |
| Procedures for misuse data |  | 1 |  | 1 | 1 |  |  |  |
| User data control |  |  | 1 |  | 1 | 1 | 1 | 1 |
| User privacy right |  |  | 1 |  | 1 | 1 | 1 | 1 |
| Data retention |  | 1 | 1 | 1 | 1 |  | 1 | 1 |
| Security features | 1 | 1 | 1 | 1 |  |  | 1 | 1 |
| Data use |  |  | 1 | 1 | 1 | 1 | 1 | 1 |
| Data ownership |  | 1 | 1 |  | 1 | 1 | 1 | 1 |
| Data storage |  |  | 1 | 1 | 1 |  | 1 | 1 |
| Data transfer |  |  | 1 |  | 1 |  |  |  |
| Age restriction |  |  |  | 1 | 1 |  |  | 1 |
| Jurisdictional Data Protection Laws, such as General Data Protection Regulation (GDPR) |  |  |  | 1 |  |  | 1 |  |
| Total score | 5 | 9 | 14 | 11 | 15 | 6 | 13 | 14 |
